# Trypanosoma cruzi Induces the PARP1/AP-1 Pathway for Upregulation of Metalloproteinases and Transforming Growth Factor β in Macrophages: Role in Cardiac Fibroblast Differentiation and Fibrosis in Chagas Disease

**DOI:** 10.1128/mBio.01853-20

**Published:** 2020-11-10

**Authors:** Subhadip Choudhuri, Nisha Jain Garg

**Affiliations:** a Department of Microbiology and Immunology, University of Texas Medical Branch, Galveston, Texas, USA; b Institute for Human Infections and Immunity, University of Texas Medical Branch, Galveston, Texas, USA; Saint Louis University; University of Arizona

**Keywords:** Chagas disease, PARP1/AP-1, TGF-β, *Trypanosoma cruzi*, cardiac fibrosis, metalloproteinases, profibrotic macrophages

## Abstract

Chagas disease (CD), caused by Trypanosoma cruzi, is a degenerative heart condition. In the present study, we investigated the role of poly [ADP-ribose] polymerase 1/activator protein 1 (PARP1/AP-1) in upregulation of profibrotic macrophages (Mϕ) and subsequent development of cardiac fibrosis in CD. We used *in vitro* and *in vivo* models of T. cruzi infection and chemical and genetic inhibition of *Parp1* to examine the molecular mechanisms by which Mϕ might augment profibrotic events in CD. Cultured (RAW 264.7 and THP-1) Mϕ infected with T. cruzi and primary cardiac and splenic Mϕ of chronically infected mice exhibited a significant increase in the expression, activity, and release of metalloproteinases (MMP2, MMP9, and MMP12) and the cytokine transforming growth factor β (TGF-β). Mϕ release of MMPs and TGF-β signaled the cardiac fibroblast to myofibroblast differentiation, as evidenced by a shift from S100A4 to alpha smooth muscle actin (α-SMA) expression. Incubation of infected Mϕ with MMP2 and MMP9 inhibitors resulted in 60 to 74% decline in TGF-β release, and MMP9 and PARP1 inhibitors resulted in 57 to 70% decline in Mϕ TGF-β-driven cardiac fibroblast differentiation. Likewise, histological studies showed a 12- to 16-fold increase in myocardial expression of CD68 (Mϕ marker) and its colocalization with MMP9/TGF-β, galectin-3, and vimentin in wild-type mice with CD. In comparison, chronically infected *Parp1*^−/−^ mice exhibited a >50% decline in myocardial levels of Mϕ and associated fibrosis markers. Further study showed that PARP1 synergized with c-Fos and JunB AP-1 family members for transcriptional activation of profibrotic response after T. cruzi infection. We conclude that PARP1 inhibition offers a potential therapy for controlling the T. cruzi-driven fibroblast differentiation in CD through modulation of the Mϕ signaling of the AP-1–MMP9–TGF-β pathway.

## Abstract (precis)

Cardiomyopathy is the most important clinical manifestation of T. cruzi-driven CD. Recent studies have suggested the detrimental role of the matrix metalloproteinases MMP2 and MMP9 in extracellular matrix (ECM) degradation during cardiac remodeling in T. cruzi infection. Peripheral TGF-β levels are increased in clinically symptomatic CD patients over those in clinically asymptomatic seropositive individuals. We provide the first evidence that during T. cruzi infection, Mϕ release of MMP2 and MMP9 plays an active role in activation of TGF-β signaling of ECM remodeling and cardiac fibroblast-to-myofibroblast differentiation. We also determined that PARP1 signals c-Fos- and JunB-mediated AP-1 transcriptional activation of profibrotic gene expression and demonstrated the significance of PARP1 inhibition in controlling chronic fibrosis in Chagas disease. Our study provides a promising therapeutic approach for controlling T. cruzi-driven fibroblast differentiation in CD by PARP1 inhibitors through modulation of the Mϕ signaling of the AP-1–MMP9–TGF-β pathway.

## INTRODUCTION

Chagas disease (CD), also known as American trypanosomiasis, is caused by infection with the protozoan parasite Trypanosoma cruzi. T. cruzi is endemic to vast areas of the Western hemisphere, from Argentina to the southern parts of the United States. T. cruzi-infected individuals are found throughout the world due to their migration from areas of high T. cruzi endemicity to areas with relatively low insect transmission or no known insect transmission (reviewed in reference 1). Currently, CD affects 6 to 8 million people worldwide. Cardiomyopathy is the most important clinical manifestation of CD, affecting ∼30% of the chronically infected individuals (2).

The pathology of CD is complex. Acute replication of T. cruzi in tissues is invariably associated with intense infiltration of mononuclear cells, including macrophages (Mϕ), neutrophils, and lymphocytes, which are capable of controlling the parasite replication and dramatically decrease the number of parasitized cells (reviewed in references 3 and 4). However, the parasite is not eliminated by the innate and adaptive immune systems, and low-grade parasite persistence associated with ongoing inflammatory reactions causes scarring of the tissues and ultimately leads to gross cardiac pathology (2). The classic pathological features of chronic Chagas cardiomyopathy include low-grade myocarditis accompanied by myocytolysis, myofiber hypertrophy, and interstitial fibrosis (5). The chronic remodeling changes are often associated with a focal or diffused inflammatory infiltrate (6), though a causal relationship between specific types of cells in the inflammatory infiltrate and myocardial fibrosis and remodeling in chronic CD has not been established.

The extracellular matrix (ECM) network provides structural support to the heart. This network is mainly formed by thick fibers of type I collagen, which provide tensile strength, and thin fibers of type III collagen, which provide elasticity, and together these facilitate transmission of systolic force (7). In addition to collagens, glycoproteins, glycosaminoglycans (hyaluronan), and proteoglycans also form the cardiac ECM (8). Cardiac fibrosis is illustrated by net accumulation of ECM proteins in the myocardium and results in both systolic and diastolic dysfunctions (9). Though this process is not fully understood, it is conceivable that following injury by T. cruzi invasion and replication, activation of the latent growth factors, like transforming growth factor beta (TGF-β1 to -β3) and proteases in the cardiac ECM as well as in the tissue-resident and infiltrating Mϕ that phagocytize parasite and remove cell debris, can trigger a fibrotic response. As the dead cells and matrix are removed and the inflammatory response resolves with control of the parasite, an overall proliferative phase with replacement of cardiomyocytes with fibroblasts, myofibroblasts, and infiltration of other cells, resulting in cardiac remodeling, would ensue. Cellular and molecular pathways that contribute to ventricular remodeling, with presentation of hypertrophy initially and with apoptosis of fibroblasts and vascular and other cells, resulting in dilation of ventricles, are not fully understood in CD.

Recent studies have identified the role of matrix metalloproteinases (MMP2 and MMP9) in ECM degradation during cardiac remodeling in T. cruzi infection (reviewed in reference 10) and suggested that MMP2 and MMP9 together can be used to predict the clinical evolution of cardiac form of CD (11). There are many cell types, including the infiltrating neutrophils and cardiac tissue-resident Mϕ, that can produce MMPs; however, current literature has not addressed the role of Mϕ in signaling tissue fibrosis in progressive CD.

In this study, we aimed to evaluate whether acute or chronic T. cruzi infection induces Mϕ expression and release of active MMPs and whether Mϕ MMPs instigate profibrotic events in the heart. In this context, we investigated whether Mϕ TGF-β activation is dependent on MMPs and causes fibroblasts to undergo myofibroblast differentiation during T. cruzi infection and chronic disease development. We recently documented that PARP1 [poly(ADP-ribose) polymerase 1], through signaling NF-κB transcriptional activation, contributes to proinflammatory activation of Mϕ (12). We therefore also determined PARP1’s role in regulation of Mϕ and cardiac fibrosis in CD. For this, we used cultured Mϕ and primary Mϕ isolated from bone marrow (BM) of wild-type (WT) and *Parp1*^−/−^ mice and employed classical approaches to evaluate the PARP1 signaling of transcriptional programs relevant to profibrotic Mϕ response during T. cruzi infection.

## RESULTS

### Macrophages express and release MMPs in response to T. cruzi infection.

**FIG 1:**
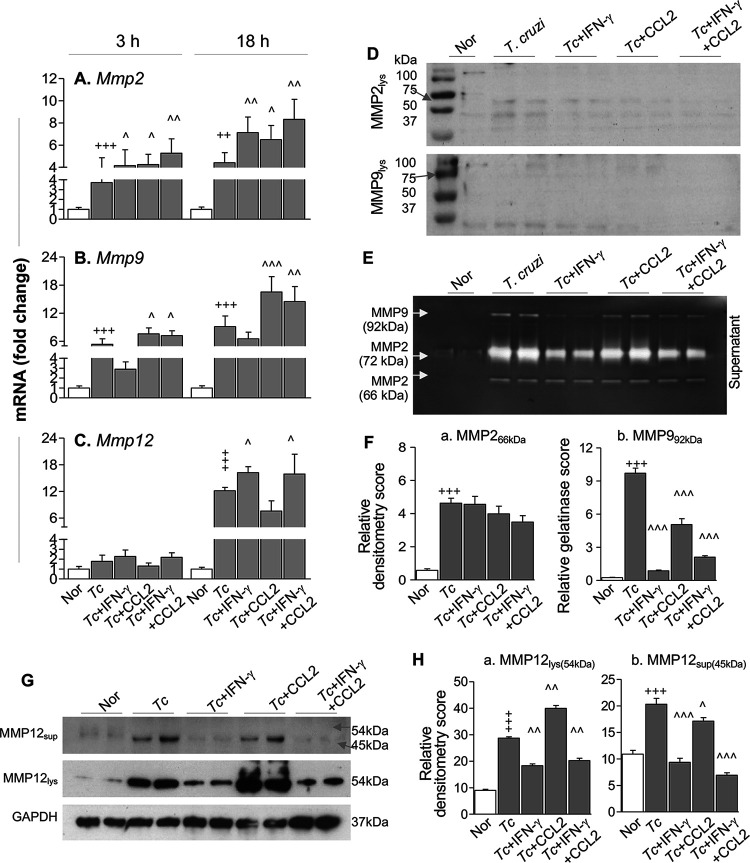
Macrophages express and release metalloproteinases in response to T. cruzi infection. RAW 264.7 Mϕ were incubated with medium or T. cruzi (*Tc*) trypomastigotes (cell-to-parasite ratio, 1:3) in the presence and absence of 20 ng/ml IFN-γ and 10 ng/ml CCL2 for 0 to 24 h. (A to C) Real-time RT-qPCR evaluation of mRNA levels for *Mmp2*, *Mmp9*, and *Mmp12* (normalized to *Gapdh*). (D) Western blot images for MMP2 and MMP9 in Mϕ cell homogenates. Cells were incubated with T. cruzi trypomastigotes (cell-to-parasite ratio, 1:3) in the presence and absence of 20 ng/ml IFN-γ and 10 ng/ml CCL2 for 24 h. (E and F) Cell supernatants were collected after 24 h of incubation, concentrated 10-fold, and used for MMP2 (pro- form, 72 kDa; cleaved form, 66 kDa) and MMP9 (pro- form, 92 kDa) zymography. Bar graphs (F) show relative gelatin degradation scores. (G and H) Western blot images for MMP12 (pro- form, 54 kDa; cleaved form, 45 kDa [G]) and relative densitometry scores for MMP12 (normalized to GAPDH [H]) in cell homogenates and supernatants (obtained after 24 h of incubation). Data in bar graphs are means and SD and are representative of two independent experiments with two biological replicates per treatment per experiment (triplicate observations per sample for RT-qPCR analysis and duplicate observations per sample for gelatin zymography and Western blot analyses). Significance is annotated as follows: +, control versus infected (Student’s two-tailed *t* test); ^, infected versus infected treated (one-way analysis of variance [ANOVA] followed by Tukey’s *post hoc* correction test). ^, *P* ≤ 0.05; ++ and ^̂, *P* ≤ 0.01; +++ and ^̂̂, *P* ≤ 0.001. Nor, normal (no infection).

We first evaluated the mRNA levels for matrix metalloproteinases (MMPs) in RAW 264.7 Mϕ infected with T. cruzi. For this, Mϕ were incubated with T. cruzi (with or without gamma interferon [IFN-γ] and C-C motif chemokine ligand 2 [CCL2], individually or in combination) for 3 h and 18 h and analyzed by reverse transcription-quantitative PCR (RT-qPCR). We included IFN-γ and CCL2 because these molecules are detected in the serum of infected mice and humans (13, 14), IFN-γ is shown to regulate MMPs through suppression of the ATF-3/AP-1 transcription program in lipopolysaccharide (LPS)-treated Mϕ (15), while CCL2 is shown to enhance MMP9 expression (16). RAW Mϕ infected with T. cruzi (versus noninfected Mϕ) exhibited 2.7- to 3.4-fold and 4.3- to 8.1-fold increases in mRNA levels for *Mmp2* and *Mmp9* (which encode gelatinases), respectively, and the maximal increase in *Mmp* mRNAs was observed at 18 h (Fig. 1A and B) (*P* < 0.001). Further, we noted an 11.1-fold increase in mRNA for *Mmp12* (which encodes macrophage metalloelastase) at 18 h (Fig. 1C) (*P* < 0.001) and a 2.3- to 5.5-fold increase in mRNA for *Mmp13* (which encodes collagenase 3) at 3 h and 18 h (Fig. S1A) (*P* < 0.001). Coincubation with IFN-γ and CCL2 had no significant effects on T. cruzi-induced mRNA levels for either of the MMPs at 3 h (Fig. 1A to C; Fig. S1A). At 18 h, IFN-γ and CCL2 either had no effect or elicited 0.15- to 0.9-fold further increases in *Mmp2*, *Mmp9*, and *Mmp12* mRNA levels in infected Mϕ (Fig. 1A to C) (*P* < 0.05). IFN-γ and CCL2 had an overall suppressive effect on T. cruzi-induced increases in *Mmp13* mRNA in Mϕ (Fig. S1A) (*P* < 0.05).

10.1128/mBio.01853-20.2 FIG S1 (A) Macrophages express *Mmp13* in response to T. cruzi infection. RAW 264.7 Mϕ were incubated for 3 h and 18 h with medium only or with T. cruzi trypomastigotes (cell-to-parasite ratio, 1:3) in the presence and absence of 20 ng/ml IFN-γ and 10 ng/ml CCL2 (individually or in combination). RT-qPCR was performed to measure mRNA for *Mmp13* (normalized to *Gapdh*). (B and C) Macrophage MMP12 expression in response to T. cruzi infection. RAW 264.7 Mϕ were incubated as described above for 48 h. Cell homogenates and supernatants (concentrated 10-fold) were analyzed by Western blotting with anti-MMP12 antibody. (B) Representative Western blot images for MMP12 (pro- form, 54 kDa; cleaved form, 45 kDa) and GAPDH (loading control). (C) Densitometry analysis of MMP12 levels, normalized to GAPDH. Data are means and SD and derived from two independent experiments consisting of two biological replicates per treatment per experiment (triplicate observations per sample for RT-qPCR and duplicate observations per sample for Western blotting). Significance is annotated as follow: +, control versus infected (Student’s two tailed t test); ^, infected versus infected and treated (one-way ANOVA followed by Tukey’s *post hoc* correction test). ^, *P* ≤ 0.05; ^̂, *P* ≤ 0.01; +++ and ^̂̂, *P* ≤ 0.001. Download FIG S1, TIF file, 0.3 MB. Copyright © 2020 Choudhuri and Garg. 2020 Choudhuri and Garg. This content is distributed under the terms of the Creative Commons Attribution 4.0 International license.

Western blot analysis and gelatin zymography were performed to assess the protein levels and activity of MMPs in infected Mϕ. Western blotting showed no significant changes in the intracellular levels of MMP2 or MMP9 in RAW Mϕ at 24 h postinfection (with or without IFN-γ and CCL2) (Fig. 1D). Instead, spent medium of infected (versus noninfected) Mϕ at 24 h showed a 7- to 36-fold increase in MMP2 (66-kDa cleaved form)- and MMP9 (92 kDa)-mediated gelatin degradation (Fig. 1E and F) (*P* < 0.001), determined by gelatin zymography. Coincubation with IFN-γ and CCL2 for 24 h had nonsignificant effects on MMP2 release (Fig. 1F, panel a) and an overall suppressive effect on MMP9 release (Fig. 1F, panel b) (*P* < 0.001) by Mϕ infected with T. cruzi. No increase in intracellular levels or release of active MMP2 and MMP9 by Mϕ was observed at 48 h postinfection.

MMP12 degrades soluble and insoluble elastin. We noted 2-fold and 9-fold increases in pro-MMP12 (54 kDa) and cleaved MMP12 (45 kDa) levels in cell lysates and spent medium, respectively, of infected (versus noninfected) Mϕ at 24 h (Fig. 1G and H) (*P* < 0.001). Coincubation with IFN-γ and CCL2 had nonsignificant or suppressive effects on T. cruzi-induced MMP12 levels at 24 h (Fig. 1G and H). By 48 h, intracellular levels of MMP12 were decreased in infected (with or without IFN-γ) Mϕ, while a 1.35-fold increase in secreted MMP12 was observed only when infected Mϕ were incubated with IFN-γ and CCL2 (Fig. S1B and C). Together, the results presented in Fig. 1 and Fig. S1 suggest that T. cruzi infection of Mϕ elicits a significant increase in the expression of MMP2, MMP9, and MMP12 metalloproteinases that is predominantly independent of the presence of IFN-γ and CCL2. Most of the MMP2 and MMP9 that exhibited gelatinase activity and the cleaved form of MMP12 were released from infected Mϕ within 24 h postinfection.

### Macrophage production of metalloproteinases and TGF-β in Chagas disease.

**FIG 2:**
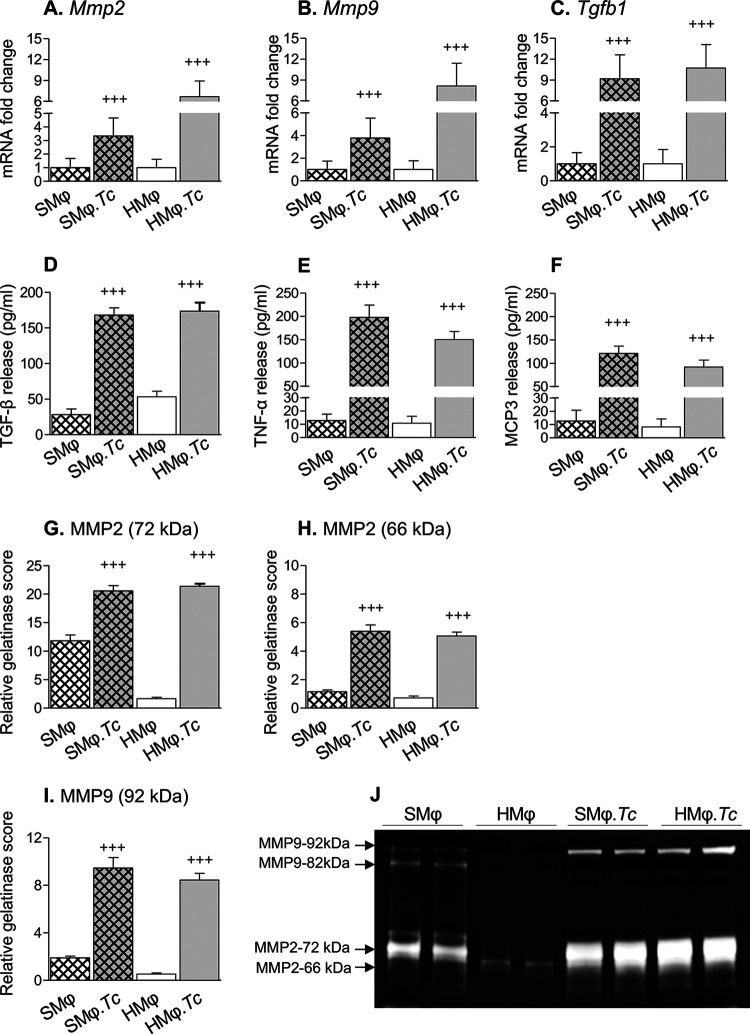
Profibrotic response of splenic and cardiac macrophages in CD-infected mice. Mice were infected with T. cruzi (10,000 parasites per mouse) and euthanized at 150 days postinfection (controls had no infection). Spleen (SMϕ) and heart (HMϕ) macrophages were isolated using CD11b^+^ magnetic beads and incubated *in vitro* for 24 h. (A to C) Real-time RT-qPCR evaluation of mRNA levels. (D to F) TGF-β, TNF-α, and MCP3 release in supernatants, determined by an ELISA. (G to J) Gelatin zymography for MMP2 and MMP9 in culture supernatants of splenic and heart Mϕ (J) and relative gelatin degradation scores for pro-MMP2 (G), cleaved MMP2 (H), and pro-MMP9 (I). Data (means and standard deviation [SD]) are representative of two independent experiments with two biological replicates per treatment (triplicate and duplicate observations per sample for RT-qPCR and zymography, respectively). Significance was calculated by Student’s two-tailed *t* test or a nonparametric Mann-Whitney U test. +++, *P* ≤ 0.001.

To verify our findings from *in vitro* studies (Fig. 1) and determine if profibrotic Mϕ are stimulated in CD, we used a well-established mouse model of T. cruzi infection (17). Mice were euthanized at 150 days postinfection, which corresponds to the chronic disease phase. The splenic and cardiac CD11b^+^ Mϕ of infected and noninfected (control) mice were isolated and incubated for 24 h in complete medium, and cells and supernatants were used for various studies. We detected 2.3-fold, 2.8-fold, and 8.1-fold increases in *Mmp2*, *Mmp9*, and *Tgfb1* mRNA levels, respectively, in splenic Mϕ of CD-infected (versus control) mice and 5.6-fold, 7.2-fold, and 9.7-fold increases in *Mmp2*, *Mmp9*, and *Tgfb1* mRNA levels, respectively, in myocardial Mϕ of chronically infected (versus noninfected) mice (Fig. 2A to C) (*P* < 0.001). Splenic and cardiac Mϕ of Chagas (versus control) mice also exhibited 5- to 14.2-fold and 2.3- to 10.2-fold increases in TGF-β, tumor necrosis factor alpha (TNF-α), and monocyte-chemotactic protein 3 (MCP3) release, respectively, after 24 h of incubation (Fig. 2D to F) (*P* < 0.001). Gelatin zymography showed 0.9-fold, 3.7-fold, and 4.0-fold increases in the gelatin degradation score for pro-MMP2 (72 kDa), cleaved MMP2 (66 kDa), and MMP9 (92 kDa), respectively, produced by splenic CD11b^+^ Mϕ of chronically infected (versus noninfected) mice (Fig. 2G to J) (*P* < 0.001). Likewise, we observed 12-fold, 6.1-fold, and 15.0-fold increase in the gelatin degradation score of pro-MMP2 and cleaved MMP2 and MMP9 (92 kDa), respectively, released by heart CD11b^+^ Mϕ of chronically infected (versus noninfected) mice (Fig. 2G and H) (*P* < 0.001). Together, these results show a profibrotic Mϕ response in chronic CD that is evidenced by an increase in the expression and release of active forms of MMP2 and MMP9 by splenic and heart Mϕ of CD-infected (versus noninfected) mice. The production of TGF-β by splenic and heart Mϕ provides further support for their profibrotic role in CD.

### MMP2 and MMP9 promote TGF-β activation in macrophages infected with T. cruzi.

**FIG 3:**
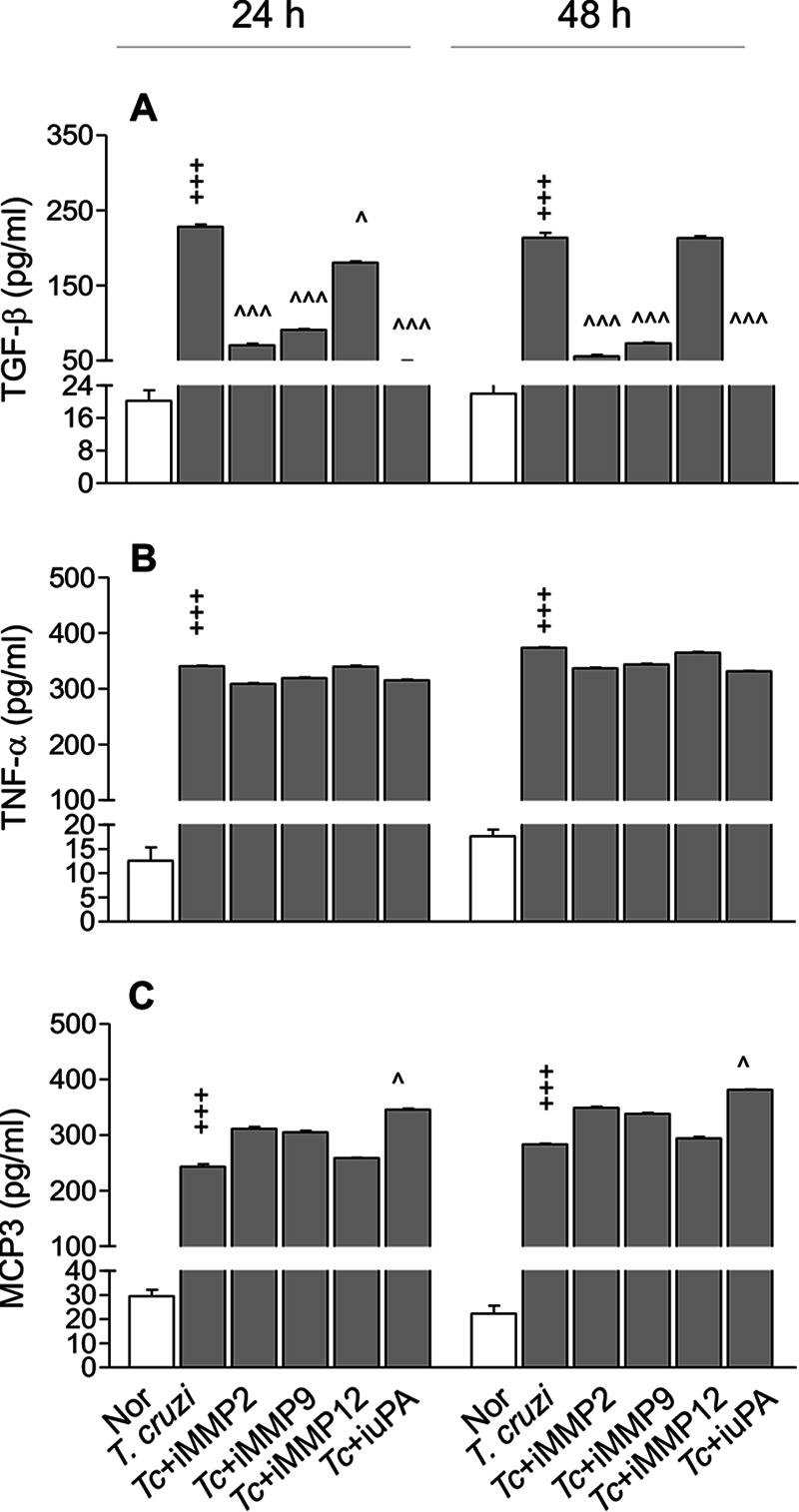
MMP2 and MMP9 are required for TGF-β production by Mϕ in response to T. cruzi infection. RAW 264.7 Mϕ were incubated with T. cruzi trypomastigotes in the presence and absence of 10 μM ARP-100 (which inhibits MMP2), 10 μM MMP9 inhibitor-1 (which inhibits MMP9), 10 μM MMP408 (which inhibits MMP12), or 10 μM uk122 (which inhibits urokinase-type plasminogen activator [uPA]). Cell culture supernatants were used for the detection of TGF-β (A), TNF-α (B), and MCP3/CCL7 (C) by an ELISA. Data are representative of two independent experiments (three biological replicates per treatment and duplicate observations per sample) and are means and SD. ^, *P* ≤ 0.05; +++ and ^̂̂, *P* ≤ 0.001 (+, noninfected versus infected by Student’s two-tailed *t* test or nonparametric Mann-Whitney U test; ^, infected versus infected/treated by one-way ANOVA and Tukey’s *post hoc* test).

MMP2 and MMP9 are (i) involved in degradation of ECM under normal physiologic and disease conditions (18), (ii) implicated in cell-to-cell communication independent of their ECM degradation function (19), and (iii) suggested to dock on the cell surface hyaluronic receptor CD44 to promote cleavage and activation of TGF-β (20). TGF-β, in turn, downregulates the inflammatory processes and activates cell-proliferative responses (18). To determine whether MMPs promote Mϕ’s profibrotic response by influencing TGF-β during T. cruzi infection, we incubated RAW Mϕ with T. cruzi in the presence or absence of specific inhibitors of MMP2, MMP9, MMP12, and urokinase-type plasminogen activator (uPA) (which activates MMP2 and MMP9) and monitored release of TGF-β and inflammatory cytokines/chemokines by an enzyme-linked immunosorbent assay (ELISA) at 24 h and 48 h. RAW Mϕ responded to T. cruzi infection with 9- to 10.5-fold, 20- to 27-fold, and 21- to 25-fold increases in TGF-β, TNF-α, and MCP3/CCL7 release, respectively, at 24 h and 48 h post-incubation (Fig. 3A to C) (*P* < 0.001). T. cruzi*-*induced TGF-β production was decreased by 69 to 74%, 60 to 66%, and 79 to 82% when infected Mϕ were incubated with inhibitors of MMP2, MMP9, and uPA, respectively (Fig. 3A) (*P* < 0.001). No effect of an MMP12 inhibitor on TGF-β production by infected Mϕ was observed (Fig. 3A). Likewise, no effect of any of the metalloproteinase inhibitors was observed on T. cruzi-induced TNF-α and MCP3/CCL7 production by Mϕ at any of the time points studied (Fig. 3B and 3C). Together, these results suggest that (i) Mϕ respond to T. cruzi with a potent increase in profibrotic (TGF-β) and proinflammatory (TNF-α and MCP3) responses and (ii) Mϕ activation of TGF-β, but not TNF-α and MCP3, was dependent on MMP2 and MMP9. Our observation of inhibitory effects of a uPA antagonist on TGF-β production suggests that active forms of MMP2 and MMP9 signal TGF-β activation in Mϕ infected by T. cruzi.

### Mϕ MMPs and TGF-β contribute to cardiac fibroblast differentiation.

**FIG 4:**
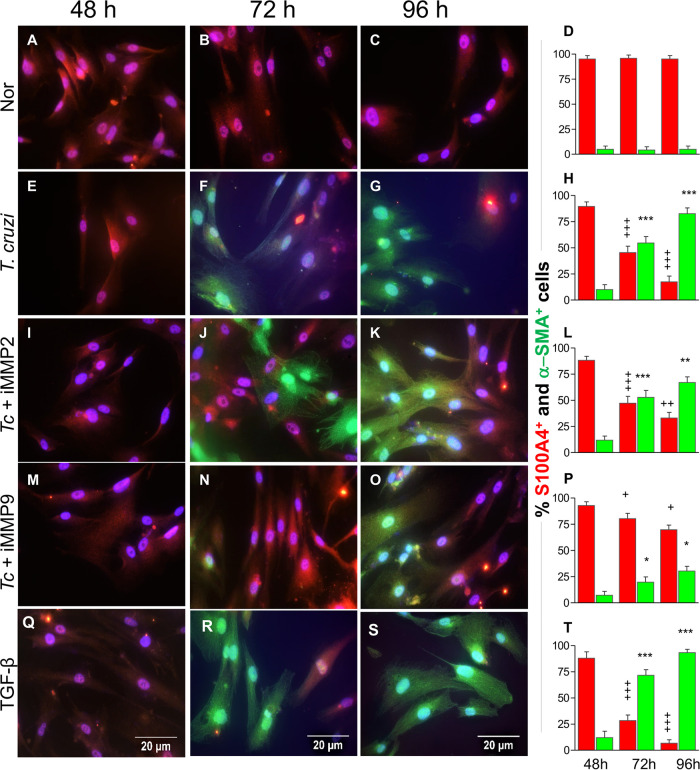
Inhibition of Mϕ MMP9 arrests TGF-β-dependent cardiac fibroblast transition to myofibroblasts in response to T. cruzi. Human THP-1 Mϕ were incubated for 24 h with T. cruzi in the presence or absence of 10 μM concentrations of inhibitors of MMP2 or MMP9. Next, human cardiac fibroblast (HCF) cells were incubated for 48 to 96 h with spent medium from THP-1 Mϕ that were noninfected (A to D), infected (E to H), or infected and treated with inhibitors of MMP2 (I to L) or MMP9 (M to P). Positive controls were HCF cells incubated with recombinant TGF-β (Q to T). Transition of HCF to myofibroblasts was determined by immunofluorescence staining for S1000A4 (red) and α-SMA (green), respectively. DAPI (4′,6-diamidino-2-phenylindole; blue) staining marks nuclei. Representative images are shown at a magnification of ×40. Bar graphs show percentages of S100A4^+^ and α-SMA^+^ HCF at 48 to 96 h post-incubation. Data (means and SD) are derived from two independent experiments, with three biological replicates per treatment, and each sample was analyzed in 9 microscopic fields. Significance comparing the expression of S100A4 (+) or α-SMA (*) at different time points was calculated by repeated-measures ANOVA or *post hoc* test. + and *, *P* ≤ 0.05; ++ and **, *P* ≤ 0.01; +++ and ***, *P* ≤ 0.001.

Cardiac fibroblast differentiation to myofibroblasts and ECM remodeling are the major drivers of cardiac fibrosis in Chagas and other heart diseases. To determine if T. cruzi-induced Mϕ production of gelatinases and TGF-β stimulated cardiac fibroblast differentiation, we incubated human THP-1 Mϕ with T. cruzi in the presence and absence of inhibitors of MMP2 and MMP9 for 24 h and collected the supernatants. Next, human cardiac fibroblasts (HCF) were incubated for 48 h, 72 h, and 96 h with spent medium from infected Mϕ and subjected to immunostaining using fluorescence-tagged antibodies against S100A4 (fibroblast-specific) and alpha smooth muscle actin (α-SMA) (myofibroblast-specific) proteins. HCF incubated with medium from T. cruzi-infected Mϕ exhibited a significant differentiation to myofibroblasts, as evidenced by a gradual increase in the frequency of α-SMA^+^ cells from 10% to 82% and a decline in the frequency of S100A4^+^ cells from 89% to 17% during 48 to 96 h of incubation (Fig. 4E to H). These findings were consistent with the observations made using recombinant TGF-β1 that also led to a gradual increase in the frequency of α-SMA^+^ cells from 12% to 93% with a corresponding decline in the frequency of S100A4^+^ cells from 87% to 7% during 48 to 96 h of incubation (Fig. 4Q to T). In comparison, incubation of HCF with spent medium from noninfected Mϕ resulted in no differentiation (Fig. 4A to D). When incubated with supernatants from THP-1 Mϕ previously treated with T. cruzi and an MMP2 antagonist, HCF continued to exhibit up to 67% differentiation to an α-SMA^+^ phenotype by 96 h (Fig. 4I to L), which indicates that MMP2 has a less significant impact on cardiac fibroblast-to-myofibroblast differentiation. In contrast, incubation of HCF with supernatant of THP-1 Mϕ previously treated with T. cruzi and an MMP9 antagonist led to only a 30% increase in the frequency of α-SMA^+^ cells, while up to 70% of the cells retained the S100A4-positive phenotype during the 96 h (Fig. 4M to P). Together, the results presented in Fig. 4, along with those in Fig. 3, suggest that MMP9 plays a key role in the release of functional TGF-β1 by Mϕ infected by T. cruzi, and Mϕ release of MMP9/TGF-β creates a microenvironment conducive to differentiation of cardiac fibroblasts to myofibroblasts.

### PARP1 signaling of profibrotic response during T. cruzi infection and Chagas disease.

**FIG 5:**
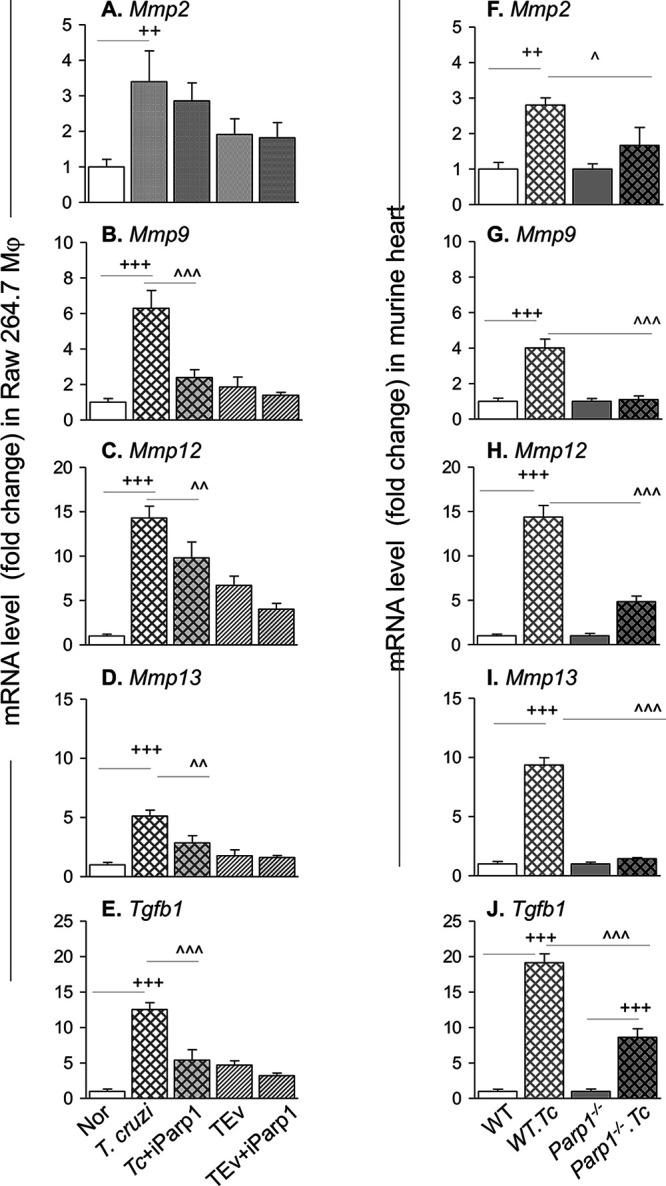
MMP and TGF-β expression in response to T. cruzi infection is PARP1 dependent. (A to E) RAW 264.7 Mϕ were incubated with T. cruzi for 72 h and extracellular vesicles (EV) released in supernatants were isolated. Next, a new batch of Mϕ was incubated for 18 h with T. cruzi or T. cruzi-induced EV (TEV) in the presence or absence of 10 μM iniparib (which inhibits PARP1). The mRNA levels for *Mmp2*, *Mmp9*, *Mmp12*, *Mmp13*, and *Tgfb1*, normalized to *Gapdh*, were measured by real-time RT-qPCR. (F to J) RT-qPCR evaluation of mRNAs in heart tissue of WT and *Parp1*^−/−^ mice euthanized at 150 days postinfection (controls had no infection). Data are representative of two independent experiments with 3 biological replicates per treatment (triplicate observations per sample). Significance is annotated as follows: +, noninfected versus infected; ^, infected versus infected with chemical or genetic deletion of PARP1 (Student’s two-tailed *t* test or nonparametric Mann-Whitney U test). ^, *P* ≤ 0.05; ++ and ^̂, *P* ≤ 0.01; +++ and ^̂̂, *P* ≤ 0.001. iParp1, inhibitor of PARP1.

We have reported that PARP1 plays a regulatory role in signaling the NF-κB-mediated proinflammatory gene expression in cardiac myocytes and Mϕ in response to T. cruzi infection (12, 21) and in Mϕ stimulated with extracellular vesicles (EV) released by infected cells (12). Since NF-κB can be involved in transcription of metalloproteinases, we determined if PARP1 also signals profibrotic gene expression during T. cruzi infection. For this, we incubated RAW Mϕ with T. cruzi in the presence or absence of 10 μM iniparib (a selective PARP1 inhibitor) for 24 h and performed RT-qPCR analysis. We also incubated Mϕ with T. cruzi-induced EV (TEV) previously purified from the culture supernatants of T. cruzi-infected Mϕ. As expected from the data in Fig. 2, Mϕ infected with T. cruzi (versus noninfected) showed 2.5-fold, 5.3-fold, 13.3-fold, 4.1-fold, and 12.5-fold increases in the mRNA levels for *Mmp2*, *Mmp9*, *Mmp12*, *Mmp13*, and *Tgfb1*, respectively (Fig. 5A to E) (*P* < 0.001). Coincubation with a PARP1 inhibitor resulted in 16%, 62%, 32%, 44%, and 57% declines in T. cruzi-induced *Mmp2*, *Mmp9*, *Mmp12*, *Mmp13*, and *Tgfb1* mRNA levels, respectively, in infected Mϕ (Fig. 5A to E) (*P* < 0.01). PARP1 was also required for driving the cardiac fibroblast differentiation to myofibroblasts. This was evidenced by the findings that HCF cells incubated for 96 h with supernatants of THP-1 Mϕ previously incubated with T. cruzi and PARP1 inhibitor exhibited only a 43% increase in the frequency of α-SMA^+^ cells (Fig. S2A to D). In comparison, up to 83% of the HCF had gained the α-SMA^+^ phenotype when incubated with supernatants of THP-1 Mϕ previously incubated with T. cruzi only (Fig. 4E to H). We observed no changes in *Mmp3* and *Mmp8* mRNA levels in Mϕ incubated with T. cruzi (with or without PARP1 inhibitor) (Fig. S2E and F) compared to matched controls. Likewise, incubation with TEV (with or without PARP1 inhibitor) resulted in no significant increase in mRNA expression for any of the metalloproteinases and TGF-β compared to that noted in normal controls (Fig. 5A to E; Fig. S2E and F).

10.1128/mBio.01853-20.3 FIG S2 (A to D) PARP1 role in cardiac fibroblast-to-myofibroblast differentiation. Human THP-1 monocytes were differentiated to Mϕ by using 50-ng/ml phorbol 12-myristate 13-acetate. THP-1 Mϕ were incubated with T. cruzi trypomastigotes in the presence of 10 μM iniparib (which inhibits PARP1) for 24 h. Next, human cardiac fibroblasts (HCF) were incubated for 48 h, 72 h, and 96 h with spent medium from infected, treated THP-1 Mϕ. Transition of HCF to myofibroblasts was determined by immunofluorescence staining for S100A4 (red) and α-SMA (green), respectively. Cells were stained with DAPI (blue) to mark the nuclei. Representative images in panels A to C are shown at a magnification of ×40. The bar graph (H) shows percentages of S100A4^+^ and α-SMA^+^ HCF at 48 to 96 h after incubation with spent medium from infected, iPARP1-treated Mϕ. Data are means and SD and are derived from two independent experiments. Each experiment included three biological replicates per treatment, and each sample was analyzed in 9 microscopic fields at a magnification of ×40. Significance comparing the expression of S100A4 (+) or α-SMA (^) at different time points was calculated by repeated-measure ANOVA with a *post hoc* test). ++ and **, *P* ≤ 0.01; +++ and ***, *P* ≤ 0.001. (E to H) T. cruzi infection does not induce *Mmp3* and *Mmp8* expression. RAW 264.7 Mϕ were incubated with medium only or T. cruzi for 72 h. Extracellular vesicles (EV) released in supernatants were isolated as described in Materials and Methods. Next, cultured Mϕ were incubated with T. cruzi trypomastigotes or T. cruzi-induced EV (TEV) in the presence or absence of 10 μm iniparib for 18 h. The mRNA levels for *Mmp3* and *Mmp8*, normalized to *Gapdh*, were measured by RT-qPCR (E and F). Mice (WT and *Parp1*^−/−^; 3 per group) were infected with T. cruzi (10,000 parasites per mouse) and euthanized at 150 days postinfection (controls had no infection). Total RNA was extracted from heart tissues, and *Mmp3* and *Mmp8* mRNAs were measured by RT-qPCR (G and H). Data are means and SD. In panels E and F, mean values are derived from two independent experiments with two biological replicates per treatment per experiment and triplicate observations per sample. In panels G and H, data are representative of 3 biological replicates with triplicate observation per sample. Student’s two-tailed *t* test or the nonparametric Mann-Whitney U test was applied, and a *P* value of <0.05 was considered the minimum level of significance. Download FIG S2, TIF file, 0.9 MB. Copyright © 2020 Choudhuri and Garg. 2020 Choudhuri and Garg. This content is distributed under the terms of the Creative Commons Attribution 4.0 International license.

We also examined the *in vivo* effects of PARP1 on the profibrotic gene expression profile in CD. These data showed 1.8-fold, 3-fold, 13.3-fold, 8.3-fold, and 18-fold increases in mRNA levels for *Mmp2*, *Mmp9*, *Mmp12*, *Mmp13*, and *Tgfb1*, respectively, in the heart tissues of chronically infected (versus noninfected) WT mice (Fig. 5F to J) (*P* < 0.01). Interestingly, chronically infected *Parp1*^−/−^ mice, in comparison to infected WT mice, exhibited 42%, 72%, 66%, 85%, and 57% declines in mRNA levels for *Mmp2*, *Mmp9*, *Mmp12*, *Mmp13*, and *Tgfb1*, respectively (Fig. 5F to J) (*P* < 0.05). We observed no changes in *Mmp3* and *Mmp8* mRNA levels in the heart tissues of chronically infected WT or *Parp1*^−/−^ mice (Fig. S2G and H) compared to matched controls.

Together, the results presented in Fig. 5 and Fig. S2 suggest that (i) PARP1 is required to activate the profibrotic transcriptional profile in Mϕ infected by T. cruzi and in heart tissues of Chagas mice and (ii) chemical inhibition or genetic deletion of *Parp1* was beneficial in arresting Mϕ profibrotic gene expression and consequently TGF-β-driven cardiac fibroblast-to-myofibroblast differentiation in response to T. cruzi infection.

### Tissue infiltration of profibrotic macrophages in Chagas mice (with and without PARP1).

**FIG 6:**
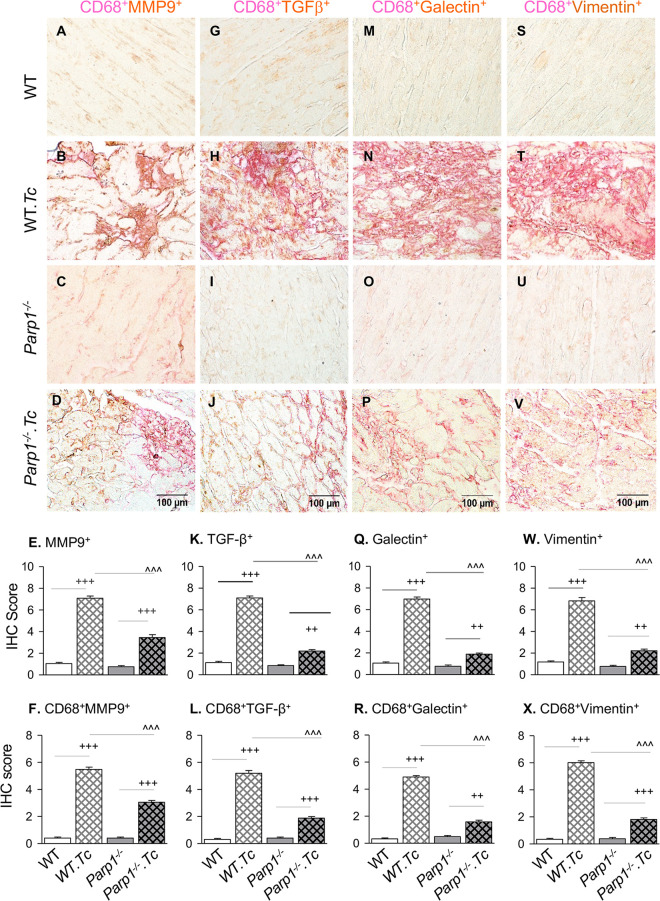
Profibrotic macrophages and tissue fibrosis in myocardium of CD-infected mice (with and without PARP1). Mice (WT and *Parp1*^−/−^) were challenged with T. cruzi and euthanized at 150 days postinfection. Paraffin-embedded left ventricular tissue sections were subjected to immunohistochemical staining. Representative images show colocalization of CD68 (magenta, Mϕ marker) with MMP9 (A to D), TGF-β (G to J), galectin (M to P), and vimentin (S to V) (magnification, ×60). Semiquantitative immunohistochemistry scores for tissue levels of MMP9, TGF-β, galectin, and vimentin (E, K, Q, and W, respectively) and CD68^+^ Mϕ colocalized with MMP9, TGF-β, galectin, and vimentin (F, L, R, and X, respectively) are shown. Bar graphs show means and SD (3 mice per group, two tissue sections per mouse, 9 microscopic fields per tissue section; magnification, ×20). Significance was calculated by Student’s two-tailed *t* test or nonparametric Mann-Whitney U *t* test and is annotated as follows: +, WT versus WT T. cruzi-infected or *Parp1*^−/−^ versus *Parp1*^−/−^ T. cruzi-infected; ^, WT T. cruzi-infected versus *Parp1*^−/−^ T. cruzi-infected. ++, *P* ≤ 0.01; +++ and ^̂̂, *P* < 0.001).

Next, we focused on determining the *in vivo* role of PARP1 and Mϕ in driving cardiac fibrosis by immunohistochemical staining of the heart tissue sections of chronically infected (and control) mice (Fig. 6). Semiquantitative scoring of immunostaining data showed that myocardial infiltration of CD68^+^ (classical Mϕ marker) cells was increased by 13-fold and 5.5-fold in chronically infected WT and *Parp1*^−/−^ (versus matched control) mice, respectively (Fig. S3) (*P* < 0.001). Myocardial frequencies of MMP9^+^ and CD68^+^ MMP9^+^ cells were increased by 6-fold and 12-fold, respectively, in WT T. cruzi-infected (versus noninfected) mice (compare Fig. 6A with Fig. 6B, E, and F), whereas *Parp1*^−/−^ T. cruzi-infected (versus WT T. cruzi-infected) mice exhibited a 45 to 50% decline in myocardial MMP9^+^ and CD68^+^ MMP9^+^ staining (compare Fig. 6B with Fig. 6D, E, and F) (*P* < 0.001). Likewise, we observed in WT T. cruzi-infected (versus noninfected) mice 4.8- to 5.4-fold increases in myocardial frequency of TGF-β^+^ (Fig. 6G, H and K), galectin-3^+^ (Fig. 6M, N and Q), and vimentin^+^ (Fig. 6S, T and W) signal and 14- to 16-fold increases in colocalization of these molecules with CD68, which indicated the role of Mϕ in driving the profibrotic (CD68^+^ TGF-β^+^) (Fig. 6L), fibrotic (CD68^+^ galectin-3^+^) (Fig. 6R), and fibroblast-to-myofibroblast differentiation (CD68^+^ vimentin^+^) profiles in CD (Fig. 6X) (*P* < 0.001 for all). In comparison to WT T. cruzi-infected mice, chronically infected *Parp1*^−/−^ mice exhibited a 68 to 73% decline in TGF-β^+^, galectin-3^+^, and vimentin^+^ myocardial staining and a 65 to 70% decline in CD68^+^ TGF-β^+^, CD68^+^ galectin-3^+^, and CD68^+^ vimentin^+^ colocalization staining (Fig. 6G to X) (*P* < 0.001). These results suggest that PARP1 regulates myocardial profibrotic response in chronic Chagas disease and that PARP1 depletion is beneficial in arresting Mϕ MMP9/TGF-β-driven cardiac fibroblast differentiation to myofibroblasts and tissue fibrosis in chronic Chagas disease-affected hearts.

10.1128/mBio.01853-20.4 FIG S3 Myocardial CD68^+^ macrophage profile in Chagas disease (with and without PARP1). Mice (WT and *Parp1*^−/−^) were challenged with T. cruzi (10,000 parasites per mouse) and euthanized at 150 days postinfection. Paraffin-embedded left ventricular heart tissue sections (5 μM) from noninfected and infected mice were subjected to immunohistochemical staining. Myocardial levels of CD68, presented as semiquantitative immunohistochemistry quick scores (means and SD), were derived from evaluating three mice per group (two tissue sections per mouse, 9 microscopic fields per tissue section; magnification, ×20). Significance is annotated as follows: +++, WT uninfected versus WT T. cruzi-infected mice or *Parp1*^−/−^ uninfected versus *Parp1*^−/−^ T. cruzi-infected mice (*P* < 0.001); ^̂̂, WT T. cruzi-infected mice versus *Parp1*^−/−^ T. cruzi-infected mice (*P* < 0.001). Student’s two-tailed *t* test or a nonparametric Mann-Whitney U test was done for statistical comparison of the variables. Download FIG S3, TIF file, 0.1 MB. Copyright © 2020 Choudhuri and Garg. 2020 Choudhuri and Garg. This content is distributed under the terms of the Creative Commons Attribution 4.0 International license.

### Transcriptional activation of profibrotic gene expression in Mϕ infected by T. cruzi (with or without PARP1).

**FIG 7:**
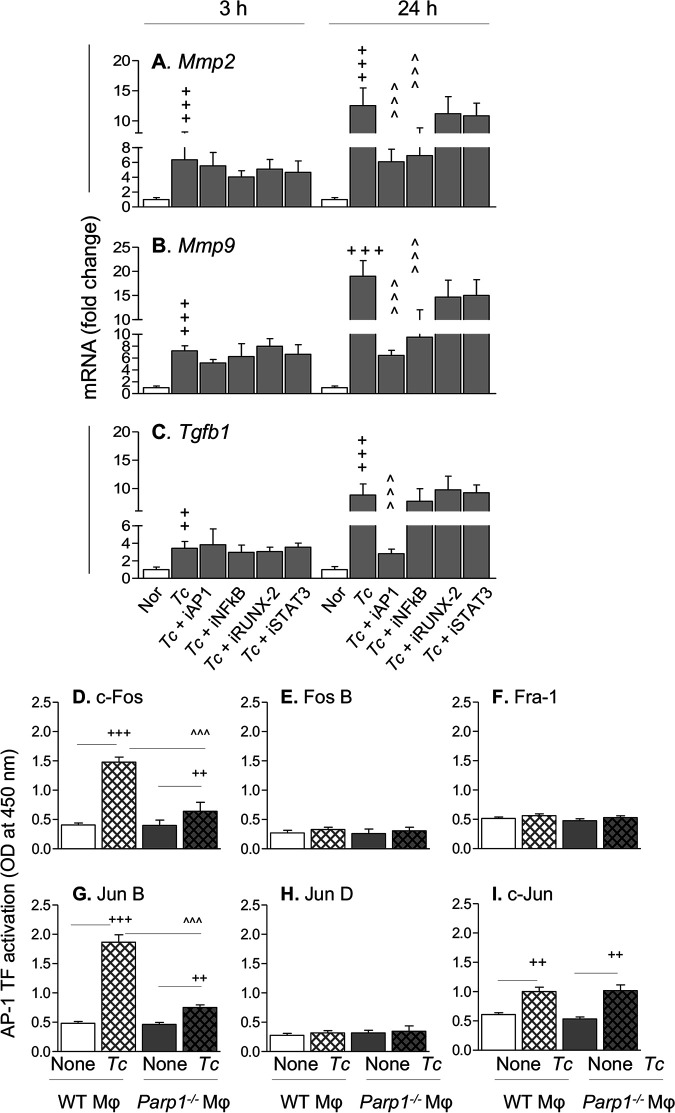
PARP1 induces T. cruzi-mediated fibrotic response through transcriptional activation of AP-1. (A to C) RAW 264.7 Mϕ were incubated with T. cruzi in the presence and absence of 10 μM concentrations of specific inhibitors of NF-κB, AP-1, RUNX-2, and STAT3. Results of real-time RT-qPCR for *Mmp2*, *Mmp9*, and *Tgfb1* (normalized to *Gapdh*) are shown. (D to I) BM-derived primary Mϕ were infected with T. cruzi for 24 h, and nuclear fractions were used to evaluate c-Fos (D), Fos B (E), Fra-1 (F), JunB (G), JunD (H), and c-Jun (I) by using AP-1 transcription factor family ELISA. Data (means and SD) are representative of two independent experiments, 2 or 3 biological replicates per treatment, and 2 or 3 observations per sample. Significance is annotated as follows: +, noninfected versus infected (Student’s two-tailed *t* test or nonparametric Mann-Whitney U test); ^, infected versus infected and treated (one-way ANOVA followed by Tukey’s *post hoc* correction test). ++, *P* ≤ 0.01; +++ and ^̂̂, *P* ≤ 0.001).

Finally, we examined transcriptional regulation of metalloproteinases in Mϕ. Genome-wide studies have identified transcription binding sites for activator protein 1 (AP-1), AP-2, NF-κB, RUNX-2, and STAT-3 in promoter sequences of genes encoding metalloproteinases, especially MMP2 and MMP9, and TGF-β (22). To determine which of these transcription factors might be involved in upregulation of the profibrotic response to T. cruzi, we infected RAW Mϕ with T. cruzi, incubated them in the presence and absence of specific antagonists of transcription factors, and monitored gene expression by RT-qPCR at 3 h and 24 h. T. cruzi induced a 3- to 7-fold increase in *Mmp2*, *Mmp9*, and *Tgfb1* mRNA levels that was not significantly changed in the presence of any of the inhibitors at 3 h (Fig. 7A to C). By 24 h, we noted an 8- to 20-fold increase in *Mmp2*, *Mmp9*, and *Tgfb1* mRNA levels in infected (versus control) Mϕ (Fig. 7A to C). Coincubation with an AP-1 inhibitor resulted in 52%, 66.5%, and 68% declines in T. cruzi-induced *Mmp2*, *Mmp9*, and *Tgfb1* mRNAs, respectively (*P* < 0.001 for all), while NF-κB inhibition resulted in a 45 to 50% decline in *Mmp2* and *Mmp9* mRNAs (*P* < 0.001) but had no effect on T. cruzi-induced increases in *Tgfb1* mRNA levels in infected Mϕ (Fig. 7A to C). Furthermore, inhibition of RUNX2 and STAT3 transcription factors had no effect on the mRNA levels of *Mmp2*, *Mmp9*, or *Tgfb1* in infected Mϕ. These results suggest that AP-1 is involved primarily in transcriptional activation of the profibrotic response in Mϕ infected by T. cruzi.

PARP1 is shown to activate JNK and DNA binding of AP-1 in fibroblasts (23) and enhance NF-κB/AP-1 binding to MMP9 promoter sequence in diabetic retinopathy (24), though its role in transcriptional activation of the profibrotic program in Mϕ, if any, is not known. To investigate whether PARP1 is engaged in AP-1 transcriptional activation of the profibrotic response, we incubated the primary bone marrow Mϕ of WT and *Parp1*^−/−^ mice with T. cruzi trypomastigotes for 24 h and utilized the nuclear extracts for an AP-1 transcription factor family assay. We found 2.64-fold, 2.86-fold, and 0.65-fold increases in c-Fos, JunB, and c-Jun AP-1 family proteins, respectively, in primary WT Mϕ infected with T. cruzi (versus no infection; *P* < 0.001) (Fig. 7D, G, and I). *Parp1*^−/−^ Mϕ infected with T. cruzi exhibited 1.3-fold and 1.5-fold declines in c-Fos and JunB levels, respectively (Fig. 7D and 7G) (*P* < 0.001), but no decline in c-Jun level (Fig. 7I) compared to infected WT Mϕ. No activation of FosB, Fra-1, or JunD AP1 transcriptional family proteins was observed in either the WT or *Parp1*^−/−^ primary Mϕ in response to T. cruzi infection (Fig. 7E, F, and H). Together, the results presented in Fig. 7 suggest that PARP1 signals transcriptional activation of c-Fos and JunB AP-1 transcriptional family proteins to regulate the Mϕ profibrotic response to T. cruzi infection and CD.

## DISCUSSION

There is a growing body of research endorsing the role of parasite persistence in facilitating the myocardial infiltration of immune and nonimmune cells and disturbing cardiac homeostasis (reviewed in reference 2). Dilated cardiomyopathy, the pathological manifestation of chronic CD, is characterized by diffused myocarditis, ECM remodeling, and interstitial fibrosis, which cause left ventricular dysfunction (5, 6). Matrix metalloproteinases, especially MMP2 and MMP9, have emerged as modulators of cardiovascular inflammation as well as remodeling. In acute T. cruzi infection, MMP2 and MMP9 expression was associated with increased inflammation, and treatment with MMP2/MMP9 antagonists was beneficial in controlling inflammatory pathology and mortality in mice (25). In chronic CD, increased plasma levels of MMP2 predicted early cardiac remodeling in clinically asymptomatic CD, while MMP9 was identified as a risk factor for late fibrosis and severe cardiac remodeling in clinically symptomatic patients (11). By using flow cytometry evaluation of peripheral blood cells, Medeiros et al. (26) showed that intracellular MMP2 and MMP9 levels were increased in neutrophils and monocytes of seropositive patients. When cells were exposed to a T. cruzi antigenic stimulus, the frequency of MMP2- and MMP9-expressing monocytes was further increased in peripheral blood cells of clinically symptomatic patients with the cardiac form of the disease but not in the seropositive, clinically asymptomatic individuals, thus implying that immune cells may contribute to fibrosis in CD through MMP production. In the present study, we demonstrate that expression and activity of MMP2, MMP9, and MMP12 were increased in cultured and primary (splenic and cardiac) Mϕ in response to T. cruzi infection and in the myocardium of chronically infected mice (Fig. 1 and 2). The Mϕ activation of metalloproteinases by T. cruzi was not critically influenced by CCL2 and IFN-γ, as is observed in other disease models (27–29). Furthermore, T. cruzi-induced Mϕ metalloproteinases (MMP9 more than MMP2) promoted TGF-β production and human cardiac fibroblast-to-myofibroblast differentiation *in vitro* (Fig. 4). The MMP2- and MMP9-producing Mϕ were also associated with myocardial fibrosis, which presented with increased tissue distribution of TGF-β (which indicates a profibrotic response), galectin-3 (a marker of fibrotic response), and vimentin (a marker of fibroblast differentiation to myofibroblasts) in chronically infected Chagas mice (Fig. 6). Our findings endorse the importance of cardiac Mϕ in driving the MMP2/MMP9-mediated, TGF-β-dependent profibrotic-fibrotic outcomes in the heart during T. cruzi infection and CD development.

The Mϕ-specific matrix metalloproteinase, i.e., MMP12, is identified as an endogenous resolution promoting factor in the events of myocardial infarction (30), and it plays a protective role by arresting inflammatory pathways and neutrophil infiltration in various diseases (31). MMP13 is a member of the collagenase family, which degrades many types of collagens and other profibrotic molecules (32). *In vitro* studies have identified many chemokines (e.g., CCL2, -3, -5, -7, -8, -15, -16, -17, and -23) as putative MMP13 substrates (33). Studies in *Mmp13*-deficient mice have identified contradictory phenotypes, with finding of attenuated inflammation and pulmonary fibrosis after irradiation-induced injury (34), no effect on fibrotic responses and an increase in inflammatory profile after hypertoxic lung injury (35), and development of severe inflammation and lung fibrosis after bleomycin treatment (33). The findings of increased expression of *Mmp12* and *Mmp13* in Mϕ infected by T. cruzi (Fig. 5C and D) and in the heart tissue of chronically infected mice (Fig. 5H and I) in this study and in a previously published study (36) and increased MMP12 expression in infected Mϕ (Fig. 1G and H) suggest that these metalloproteinases may also play a key role in regulating pathological inflammation and fibrosis in CD. However, the mechanistic role of MMP12 and MMP13 in the context of CD was not addressed in this study or in previously published literature, and this remains to be examined in future studies.

The role of TGF-β signaling in remodeling heart is complex and not completely understood. This is because its diverse effects elicit multiple and often opposing cellular responses. In brief, the TGF-β superfamily transduces signal to downstream effectors primarily through the SMAD family of proteins, but it may also activate other signaling cascades, including extracellular signal-regulated kinase (Erk), c-Jun N-terminal kinase (JNK), TGF-β-activated kinase 1 (TAK1), and p38 mitogen-activated protein kinase (MAPK) pathways (37, 38). Depending on the time, extent, and types of surface receptors and intracellular signaling cascades engaged, TGF-β may play a crucial role in repression of the inflammatory response and mediate resolution of the inflammatory infiltrate after injuries caused by infectious or genotoxic stimuli (9). TGF-β may be cardioprotective or play a key role as a mediator of pathological hypertrophy and ventricular remodeling, depending on the extent to which it modulates the fibroblast phenotype, promotes ECM deposition by upregulating collagen and fibronectin synthesis and by decreasing matrix degradation through induction of protease inhibitors, or stimulates cardiomyocyte growth and induces interstitial fibrosis (7, 39). In CD, TGF-β is suggested to serve as a growth factor, promoting parasite replication (40), and treatment with a TGF-β receptor inhibitor decreased the parasitemia, heart damage, and mortality in infected mice (41). Patients with the clinically symptomatic cardiac form of CD exhibit higher serum levels of TGF-β than clinically asymptomatic seropositive patients, and CD patients with higher TGF-β levels present a worse clinical outcome (42, 43). Our study showed significantly higher *Tgfb1* expression in the heart tissues of chronic CD-infected mice and T. cruzi-infected Mϕ. Thus, current literature and our data corroborate the importance of TGF-β in the development and maintenance of cardiac damage in response to T. cruzi infection.

Besides its complex role, the unusual biology of TGF-β activation is intriguing. Using a 3-dimensional collagen gel model loaded with TGF-β in conjunction with fibroblasts deficient in MMP9, Kobayashi et al. (44) showed that MMP9 was required to activate TGF-β and that MMP9 deficiency reduced the TGF-β-driven responses, including activity of a *Smad3* reporter gene and production of fibronectin. Using TA3 murine mammary carcinoma cells, Yu and Stamenkovic (20) showed that MMP9 is localized on the cell surface in a CD44-dependent manner and stimulates proteolytic cleavage of TGF-β, and they proposed a novel role for MMP9 in TGF-β-dependent tumorigenesis. Activation of TGF-β1 was at least partially dependent on MMP2 activity in age-associated arterial remodeling (45). TGF-β, in turn, can inhibit MMP9 activity through the synthesis of protease inhibitors. Our data show that inhibition of MMP2, MMP9, or uPA (but not MMP12) abolished the Mϕ release of TGF-β in response to T. cruzi infection. Whether MMP2 and MMP9 influence TGF-β release through regulation of transcriptional, translational, or posttranslational activation programs in Mϕ infected by T. cruzi remains to be examined in future studies. Nonetheless, our findings provide the first evidence that during T. cruzi infection, Mϕ MMP9 plays a critical role in the activation of latent TGF-β and that this in turn regulates cardiac fibroblast-to-myofibroblast differentiation.

PARP1 is widely regarded as an intracellular DNA damage sensor. In CD, innate immune cells responding to low-grade parasite persistence and mitochondrial dysfunction are recognized as major sources of free radicals (reviewed in references 2, 46, and 47), and the resultant oxidative DNA adducts were shown to cause PARP1 hyperactivation in cardiomyocytes and heart tissue infected by T. cruzi (21, 48). PARP1 direct binding and PARP1-mediated PARylation of proteins can affect the expression of inflammation-related genes at the transcriptional and posttranscriptional levels (21), and PARP1-dependent NF-κB-mediated proinflammatory gene expression was increased in cardiac myocytes infected by T. cruzi (48). Further, we have found that T. cruzi infection and chronic CD induce release of extracellular vesicles (EV) carrying oxidized DNA of host and parasite origin. When phagocytized by Mϕ, the oxidized DNA in these EV was sensed by the cytosolic DNA response element cGAS, which synergized with PARP1 to signal NF-κB transcriptional activation (12). Our results in this study show that T. cruzi-induced EV (TEV) were not profibrotic, and invasion by live T. cruzi was required to induce the MMP/TGF-β expression in Mϕ. These findings suggest that TEV do not regulate the MMP and TGF-β expression in Mϕ by the involvement of cytosolic cGAS-PARP1 and the downstream NF-κB signaling pathway. Instead, nuclear activation of Fos and JunB AP-1 transcription family proteins, in synergy with nuclear PARP1, signaled the increase in the expression and activity of MMP2, MMP9, and TGF-β in Mϕ infected with T. cruzi (Fig. 7). This was evidenced by our findings in this study that genetic deletion or chemical inhibition of PARP1 abolished the T. cruzi-induced increase in mRNAs for *Mmp2*, *Mmp9*, *Mmp12*, and *Mmp13* in Mϕ and cardiac tissue of Chagas mice and decreased the expression of MMP9, TGF-β, and profibrotic/fibrotic differentiation markers in heart tissue of CD-infected mice. Likewise, we reported previously that PJ34 (a PARP1 inhibitor) significantly improved the mitochondrial biogenesis and antioxidant capacity in the heart tissues of T. cruzi-infected mice and genetic deletion of PARP1 decreased the collagen deposition and the cardiac fibrosis and remodeling in chronically infected mice (49). Others have shown that treatment with olaparib (PARP1 inhibitor) ameliorated MMP2 and MMP9 expression in an elastase-induced mouse model of emphysema (50). Thus, based on our previous and present findings, we surmise that PARP1 plays an important role in progression of inflammation and fibrosis in CD through the engagement of cGAS-NF-κB and AP-1 signaling pathways.

MMP promoters harbor several *cis* elements, which allow the regulation of MMP gene expression by various transcriptional factors, such as AP-1, NF-κB, RUNX2, and STAT3, among others (reviewed in reference 22). PARP1 was shown to promote nuclear translocation of RelA (p65) in T. cruzi-infected cardiomyocytes (48) and to enhance NF-κB/AP-1 binding to the MMP9 promoter sequence in diabetic retinopathy (24) and JNK-dependent activation of AP-1 in murine fibroblasts (23). In this study, NF-κB only partially contributed to increases in *Mmp2* and *Mmp9* expression, and AP-1 was necessary to signal Mϕ expression of *Mmp2*, *Mmp9*, and *Tgfb1* in response to T. cruzi infection. Further, *Parp1*^−/−^ (versus control) Mϕ exhibited significantly low activity of c-Fos and JunB AP-1 transcriptional family proteins. Our findings establish that PARP1 functions as a transcriptional coregulator of c-Fos and JunB members of the AP1 family to drive the profibrotic/fibrotic response of Mϕ, and they offer novel insights into the role of PARP1 in the transcriptional regulation and release of Mϕ gelatinases and profibrotic TGF-β in Chagas disease.

In summary, we used *in vitro* and *in vivo* models of T. cruzi infection and CD to show that Mϕ MMP2 and MMP9 released during T. cruzi infection are active and shape the ECM profibrotic response by the release of active TGF-β. Chemical inhibition or genetic deletion of PARP1 arrested c-Fos- and JunB-mediated AP-1 transcriptional activation of MMP/TGF-β responses in Mϕ, and this was beneficial in arresting the T. cruzi-driven cardiac fibroblast-to-myofibroblast differentiation in chronic CD. One limitation of the present study is that *Parp1*^−/−^ mice express a shortened transcript lacking enzymatic activity in all cells and tissues, and it is possible that the observed decline in myocardial infiltration of profibrotic Mϕ and cardiac fibrosis in *Parp1*^−/−^ mice was an outcome of the cumulative effects of PARP1 on AP-1–MMP9–TGF-β signaling pathway (this study) and cGAS-NF-κB signaling of proinflammatory responses (our previous study [12]). Future studies in mice with Mϕ- and cardiomyocyte-specific knockdown of PARP1 will be needed to examine the relative importance of PARP1 signaling of cGAS-NF-κB and AP-1-MMP9-TGF-β pathways in CD pathogenesis. Nevertheless, our findings make PARP1 an attractive target for CD therapy and provide an impetus for testing the efficacy of a variety of PARP1 inhibitors in controlling chronic inflammatory infiltrate and cardiac fibrosis, the two hallmarks of CD severity, in multiple mammalian hosts.

## MATERIALS AND METHODS

Six-week-old wild-type and *Parp1*^−/−^ (both male and female) mice were infected with trypomastigotes of T. cruzi strain Sylvio X10 obtained from ATCC (10,000 parasites per mouse) and euthanized at ∼150 days postinfection. RAW 264.7 (ATCC TIB-71), THP-1, and primary murine Mϕ and human cardiac fibroblasts (HCF) were cultured by standard protocols. Myocardial and splenic Mϕ were purified using the MagniSort mouse CD11b positive-selection kit. Mϕ were incubated with T. cruzi (cell-to-parasite ratio, 1:3), extracellular vesicles (EV) released from the infected cells, and/or various reagents for the time periods stated in the figure legends.

### Gene and protein expression.

Changes in the expression of target genes was examined by real-time RT-qPCR (22). Protein levels were determined by Western blotting. Zymography was performed to examine the release of MMP2 and MMP9 using Novex 10% Zymogram Plus gels following the manufacturer’s instructions (Thermo Fisher Scientific). Images were analyzed by using ImageJ software. Quantitative ELISA was performed to monitor the release of active TGF-β1 (no. 88-8350; eBioscience), TNF-α (no. 88-7324; eBioscience), and MCP3/CCL7 (no. 205571; Abcam) in culture supernatants.

### Immunostaining.

HCF were incubated with spent cell culture supernatants of THP-1 Mϕ incubated with T. cruzi trypomastigotes and various inhibitors. Cells were sequentially incubated with anti-mouse S100A4 and rabbit anti-mouse alpha smooth muscle actin (α-SMA) primary antibodies and fluorescence-conjugated secondary antibodies, and signals were analyzed on an Olympus BX-15 fluorescence microscope equipped with Simple PCI software (12).

Tissue sections were incubated with primary antibodies against CD68, TGF-β, MMP9, galectin-3, and vimentin followed by horseradish peroxidase (HRP)- or allophycocyanin (AP)-conjugated secondary antibodies, and intensity and distribution of staining were scored as described previously (33).

### AP-1 transcriptional activity.

BM-derived primary Mϕ (WT and *Parp1*^−/−^) were incubated with T. cruzi and specific inhibitors, and AP-1 transcriptional activity was monitored using the 96-well TransAM AP-1 family transcription factor assay kit.

### Statistical analysis.

Data are presented as means and standard deviations (SD) (*P* < 0.05). Details are given in the figure legends.

Detailed materials and methods are presented in Text S1.

10.1128/mBio.01853-20.1 TEXT S1 Detailed materials and methods. Download Text S1, DOCX file, 0.04 MB. Copyright © 2020 Choudhuri and Garg. 2020 Choudhuri and Garg. This content is distributed under the terms of the Creative Commons Attribution 4.0 International license.

## References

[1] Rios L, Campos EE, Menon R, Zago MP, Garg NJ. 2020. Epidemiology and pathogenesis of fetal-transplacental transmission of *Trypanosoma cruzi* and a case for vaccine development against congenital Chagas disease. Biochim Biophys Acta Mol Basis Dis 1866:165591. doi:10.1016/j.bbadis.2019.165591. 31678160 PMC6954953

[2] Bonney KM, Luthringer DJ, Kim SA, Garg NJ, Engman DM. 2019. Pathology and pathogenesis of Chagas heart disease. Annu Rev Pathol 14:421–447. doi:10.1146/annurev-pathol-020117-043711. 30355152 PMC7373119

[3] Acevedo GR, Girard MC, Gomez KA. 2018. The unsolved jigsaw puzzle of the immune response in Chagas disease. Front Immunol 9:1929. doi:10.3389/fimmu.2018.01929. 30197647 PMC6117404

[4] Rios LE, Vazquez-Chagoyan JC, Pacheco AO, Zago MP, Garg NJ. 2019. Immunity and vaccine development efforts against *Trypanosoma cruzi*. Acta Trop 200:105168. doi:10.1016/j.actatropica.2019.105168. 31513763 PMC7409534

[5] Tanowitz HB, Machado FS, Spray DC, Friedman JM, Weiss OS, Lora JN, Nagajyothi J, Moraes DN, Garg NJ, Nunes MCP, Ribeiro ALP. 2015. Developments in the manangement of Chagas cardiomyopathy. Expert Rev Cardiovasc Ther 13:1393–1409. doi:10.1586/14779072.2015.1103648. 26496376 PMC4810774

[6] Machado FS, Dutra WO, Esper L, Gollob KJ, Teixeira MM, Factor SM, Weiss LM, Nagajyothi F, Tanowitz HB, Garg NJ. 2012. Current understanding of immunity to *Trypanosoma cruzi* infection and pathogenesis of Chagas disease. Semin Immunopathol 34:753–770. doi:10.1007/s00281-012-0351-7. 23076807 PMC3498515

[7] Frangogiannis NG. 2019. The extracellular matrix in ischemic and nonischemic heart failure. Circ Res 125:117–146. doi:10.1161/CIRCRESAHA.119.311148. 31219741 PMC6588179

[8] Mouw JK, Ou G, Weaver VM. 2014. Extracellular matrix assembly: a multiscale deconstruction. Nat Rev Mol Cell Biol 15:771–785. doi:10.1038/nrm3902. 25370693 PMC4682873

[9] Kong P, Christia P, Frangogiannis NG. 2014. The pathogenesis of cardiac fibrosis. Cell Mol Life Sci 71:549–574. doi:10.1007/s00018-013-1349-6. 23649149 PMC3769482

[10] Medeiros NI, Gomes JAS, Correa-Oliveira R. 2017. Synergic and antagonistic relationship between MMP-2 and MMP-9 with fibrosis and inflammation in Chagas' cardiomyopathy. Parasite Immunol 39:e12446. doi:10.1111/pim.12446. 28543409

[11] Medeiros NI, Gomes JAS, Fiuza JA, Sousa GR, Almeida EF, Novaes RO, Rocha VLS, Chaves AT, Dutra WO, Rocha MOC, Correa-Oliveira R. 2019. MMP-2 and MMP-9 plasma levels are potential biomarkers for indeterminate and cardiac clinical forms progression in chronic Chagas disease. Sci Rep 9:14170. doi:10.1038/s41598-019-50791-z. 31578449 PMC6775161

[12] Choudhuri S, Garg NJ. 2020. PARP1-cGAS-NFkB pathway of proinflammatory macrophage activation by extracellular vesicles released during *Trypanosoma cruzi* infection and Chagas disease. PLoS Pathog 16:e1008474. doi:10.1371/journal.ppat.1008474. 32315358 PMC7173744

[13] Talvani A, Rocha MO, Barcelos LS, Gomes YM, Ribeiro AL, Teixeira MM. 2004. Elevated concentrations of CCL2 and tumor necrosis factor-alpha in chagasic cardiomyopathy. Clin Infect Dis 38:943–950. doi:10.1086/381892. 15034825

[14] Cunha-Neto E, Dzau VJ, Allen PD, Stamatiou D, Benvenutti L, Higuchi ML, Koyama NS, Silva JS, Kalil J, Liew C-C. 2005. Cardiac gene expression profiling provides evidence for cytokinopathy as a molecular mechanism in Chagas' disease cardiomyopathy. Am J Pathol 167:305–313. doi:10.1016/S0002-9440(10)62976-8. 16049318 PMC1603558

[15] Ho HH, Antoniv TT, Ji JD, Ivashkiv LB. 2008. Lipopolysaccharide-induced expression of matrix metalloproteinases in human monocytes is suppressed by IFN-gamma via superinduction of ATF-3 and suppression of AP-1. J Immunol 181:5089–5097. doi:10.4049/jimmunol.181.7.5089. 18802113 PMC2610354

[16] Tang CH, Tsai CC. 2012. CCL2 increases MMP-9 expression and cell motility in human chondrosarcoma cells via the Ras/Raf/MEK/ERK/NF-kappaB signaling pathway. Biochem Pharmacol 83:335–344. doi:10.1016/j.bcp.2011.11.013. 22138288

[17] Gupta S, Garg NJ. 2015. A two-component DNA-prime/protein-boost vaccination strategy for eliciting long-term, protective T cell immunity against *Trypanosoma cruzi*. PLoS Pathog 11:e1004828. doi:10.1371/journal.ppat.1004828. 25951312 PMC4423834

[18] da Costa AWF, do Carmo Neto JR, Braga YLL, Silva BA, Lamounier AB, Silva BO, Dos Reis MA, de Oliveira FA, Celes MRN, Machado JR. 2019. Cardiac Chagas disease: MMPs, TIMPs, galectins, and TGF-beta as tissue remodelling players. Dis Markers 2019:3632906. doi:10.1155/2019/3632906. 31885735 PMC6899287

[19] Valiente-Alandi I, Schafer AE, Blaxall BC. 2016. Extracellular matrix-mediated cellular communication in the heart. J Mol Cell Cardiol 91:228–237. doi:10.1016/j.yjmcc.2016.01.011. 26778458 PMC4767504

[20] Yu Q, Stamenkovic I. 2000. Cell surface-localized matrix metalloproteinase-9 proteolytically activates TGF-beta and promotes tumor invasion and angiogenesis. Genes Dev 14:163–176. 10652271 PMC316345

[21] Ba X, Gupta S, Davidson M, Garg NJ. 2010. *Trypanosoma cruzi* induces ROS-PARP-1-RelA pathway for up regulation of cytokine expression in cardiomyocytes. J Biol Chem 285:11596–11606. doi:10.1074/jbc.M109.076984. 20145242 PMC2857037

[22] Fanjul-Fernandez M, Folgueras AR, Cabrera S, Lopez-Otin C. 2010. Matrix metalloproteinases: evolution, gene regulation and functional analysis in mouse models. Biochim Biophys Acta 1803:3–19. doi:10.1016/j.bbamcr.2009.07.004. 19631700

[23] Andreone TL, O'Connor M, Denenberg A, Hake PW, Zingarelli B. 2003. Poly(ADP-ribose) polymerase-1 regulates activation of activator protein-1 in murine fibroblasts. J Immunol 170:2113–2120. doi:10.4049/jimmunol.170.4.2113. 12574383

[24] Mishra M, Kowluru RA. 2017. Role of PARP-1 as a novel transcriptional regulator of MMP-9 in diabetic retinopathy. Biochim Biophys Acta Mol Basis Dis 1863:1761–1769. doi:10.1016/j.bbadis.2017.04.024. 28478229 PMC5599300

[25] Gutierrez FRS, Lalu MM, Mariano FS, Milanezi CM, Cena J, Gerlach RF, Santos JET, Torres-Dueñas D, Cunha FQ, Schulz R, Silva JS. 2008. Increased activities of cardiac matrix metalloproteinases matrix metalloproteinase (MMP)-2 and MMP-9 are associated with mortality during the acute phase of experimental *Trypanosoma cruzi* infection. J Infect Dis 197:1468–1476. doi:10.1086/587487. 18444803

[26] Medeiros NI, Fares RCG, Franco EP, Sousa GR, Mattos RT, Chaves AT, Nunes MDCP, Dutra WO, Correa-Oliveira R, Rocha MOC, Gomes JAS. 2017. Differential expression of matrix metalloproteinases 2, 9 and cytokines by neutrophils and monocytes in the clinical forms of Chagas disease. PLoS Negl Trop Dis 11:e0005284. doi:10.1371/journal.pntd.0005284. 28118356 PMC5261563

[27] Nosaka M, Ishida Y, Kimura A, Kuninaka Y, Inui M, Mukaida N, Kondo T. 2011. Absence of IFN-gamma accelerates thrombus resolution through enhanced MMP-9 and VEGF expression in mice. J Clin Invest 121:2911–2920. doi:10.1172/JCI40782. 21646723 PMC3223815

[28] Robinson SC, Scott KA, Balkwill FR. 2002. Chemokine stimulation of monocyte matrix metalloproteinase-9 requires endogenous TNF-alpha. Eur J Immunol 32:404–412. doi:10.1002/1521-4141(200202)32:2<404::AID-IMMU404>3.0.CO;2-X. 11813159

[29] Quiding-Jarbrink M, Smith DA, Bancroft GJ. 2001. Production of matrix metalloproteinases in response to mycobacterial infection. Infect Immun 69:5661–5670. doi:10.1128/iai.69.9.5661-5670.2001. 11500442 PMC98682

[30] Mouton AJ, Rivera Gonzalez OJ, Kaminski AR, Moore ET, Lindsey ML. 2018. Matrix metalloproteinase-12 as an endogenous resolution promoting factor following myocardial infarction. Pharmacol Res 137:252–258. doi:10.1016/j.phrs.2018.10.026. 30394317 PMC6239213

[31] Bellac CL, Dufour A, Krisinger MJ, Loonchanta A, Starr AE, Auf Dem Keller U, Lange PF, Goebeler V, Kappelhoff R, Butler GS, Burtnick LD, Conway EM, Roberts CR, Overall CM. 2014. Macrophage matrix metalloproteinase-12 dampens inflammation and neutrophil influx in arthritis. Cell Rep 9:618–632. doi:10.1016/j.celrep.2014.09.006. 25310974

[32] Li H, Wang D, Yuan Y, Min J. 2017. New insights on the MMP-13 regulatory network in the pathogenesis of early osteoarthritis. Arthritis Res Ther 19:248. doi:10.1186/s13075-017-1454-2. 29126436 PMC5681770

[33] Cabrera S, Maciel M, Hernández-Barrientos D, Calyeca J, Gaxiola M, Selman M, Pardo A. 2019. Delayed resolution of bleomycin-induced pulmonary fibrosis in absence of MMP13 (collagenase 3). Am J Physiol Lung Cell Mol Physiol 316:L961–L976. doi:10.1152/ajplung.00455.2017. 30785343

[34] Flechsig P, Hartenstein B, Teurich S, Dadrich M, Hauser K, Abdollahi A, Gröne H-J, Angel P, Huber PE. 2010. Loss of matrix metalloproteinase-13 attenuates murine radiation-induced pulmonary fibrosis. Int J Radiat Oncol Biol Phys 77:582–590. doi:10.1016/j.ijrobp.2009.12.043. 20457355

[35] Sen AI, Shiomi T, Okada Y, D'Armiento JM. 2010. Deficiency of matrix metalloproteinase-13 increases inflammation after acute lung injury. Exp Lung Res 36:615–624. doi:10.3109/01902148.2010.497201. 20860538 PMC3286187

[36] Lokugamage N, Choudhuri S, Davies C, Chowdhury IH, Garg NJ. 2020. Antigen-based nano-immunotherapy controls parasite persistence, inflammatory and oxidative stress, and cardiac fibrosis, the hallmarks of chronic Chagas cardiomyopathy, in a mouse model of *Trypanosoma cruzi* infection. Vaccines (Basel) 8:96. doi:10.3390/vaccines8010096. PMC7157635 32098116

[37] Parichatikanond W, Luangmonkong T, Mangmool S, Kurose H. 2020. Therapeutic targets for the treatment of cardiac fibrosis and cancer: focusing on TGF-beta signaling. Front Cardiovasc Med 7:34. doi:10.3389/fcvm.2020.00034. 32211422 PMC7075814

[38] Hu H-H, Chen D-Q, Wang Y-N, Feng Y-L, Cao G, Vaziri ND, Zhao Y-Y. 2018. New insights into TGF-beta/Smad signaling in tissue fibrosis. Chem Biol Interact 292:76–83. doi:10.1016/j.cbi.2018.07.008. 30017632

[39] Bujak M, Frangogiannis NG. 2007. The role of TGF-beta signaling in myocardial infarction and cardiac remodeling. Cardiovasc Res 74:184–195. doi:10.1016/j.cardiores.2006.10.002. 17109837 PMC1924687

[40] Ming M, Ewen ME, Pereira ME. 1995. Trypanosome invasion of mammalian cells requires activation of the TGF beta signaling pathway. Cell 82:287–296. doi:10.1016/0092-8674(95)90316-x. 7628017

[41] Waghabi MC, de Souza EM, de Oliveira GM, Keramidas M, Feige J-J, Araújo-Jorge TC, Bailly S. 2009. Pharmacological inhibition of transforming growth factor beta signaling decreases infection and prevents heart damage in acute Chagas' disease. Antimicrob Agents Chemother 53:4694–4701. doi:10.1128/AAC.00580-09. 19738024 PMC2772341

[42] Saraiva RM, Waghabi MC, Vilela MF, Madeira FS, Sperandio da Silva GM, Xavier SS, Feige JJ, Hasslocher-Moreno AM, Araujo-Jorge TC. 2013. Predictive value of transforming growth factor-beta1in Chagas disease: towards a biomarker surrogate of clinical outcome. Trans R Soc Trop Med Hyg 107:518–525. doi:10.1093/trstmh/trt050. 23787193

[43] Araújo-Jorge TC, Waghabi MC, Hasslocher-Moreno AM, Xavier SS, Higuchi MDL, Keramidas M, Bailly S, Feige J-J. 2002. Implication of transforming growth factor-beta1 in Chagas disease myocardiopathy. J Infect Dis 186:1823–1828. doi:10.1086/345882. 12447769

[44] Kobayashi T, Kim H, Liu X, Sugiura H, Kohyama T, Fang Q, Wen F-Q, Abe S, Wang X, Atkinson JJ, Shipley JM, Senior RM, Rennard SI. 2014. Matrix metalloproteinase-9 activates TGF-beta and stimulates fibroblast contraction of collagen gels. Am J Physiol Lung Cell Mol Physiol 306:L1006–L1015. doi:10.1152/ajplung.00015.2014. 24705725 PMC4042193

[45] Wang M, Zhao D, Spinetti G, Zhang J, Jiang L-Q, Pintus G, Monticone R, Lakatta EG. 2006. Matrix metalloproteinase 2 activation of transforming growth factor-beta1 (TGF-beta1) and TGF-beta1-type II receptor signaling within the aged arterial wall. Arterioscler Thromb Vasc Biol 26:1503–1509. doi:10.1161/01.ATV.0000225777.58488.f2. 16690877

[46] Lopez M, Tanowitz HB, Garg NJ. 2018. Pathogenesis of chronic Chagas disease: macrophages, mitochondria, and oxidative stress. Curr Clin Microbiol Rep 5:45–54. doi:10.1007/s40588-018-0081-2. 29868332 PMC5983038

[47] Koo SJ, Garg NJ. 2019. Metabolic programming of macrophage functions and pathogens control. Redox Biol 24:101198. doi:10.1016/j.redox.2019.101198. 31048245 PMC6488820

[48] Ba X, Garg NJ. 2011. Signaling mechanism of PARP-1 in inflammatory diseases. Am J Pathol 178:946–955. doi:10.1016/j.ajpath.2010.12.004. 21356345 PMC3069822

[49] Wen JJ, Yin YW, Garg NJ. 2018. PARP1 depletion improves mitochondrial and heart function in Chagas disease: effects on POLG dependent mtDNA maintenance. PLoS Pathog 14:e1007065. doi:10.1371/journal.ppat.1007065. 29851986 PMC5979003

[50] Dharwal V, Sandhir R, Naura AS. 2019. PARP-1 inhibition provides protection against elastase-induced emphysema by mitigating the expression of matrix metalloproteinases. Mol Cell Biochem 457:41–49. doi:10.1007/s11010-019-03510-1. 30993494

