# Supplementary figures and images for: Trypanosoma cruzi Induces the PARP1/AP-1 Pathway for Upregulation of Metalloproteinases and Transforming Growth Factor β in Macrophages: Role in Cardiac Fibroblast Differentiation and Fibrosis in Chagas Disease
Source: mBio. 2020 Nov 10;11(6):e01853-20. doi: 10.1128/mBio.01853-20 (PMC7667027; doi:10.1128/mBio.01853-20)

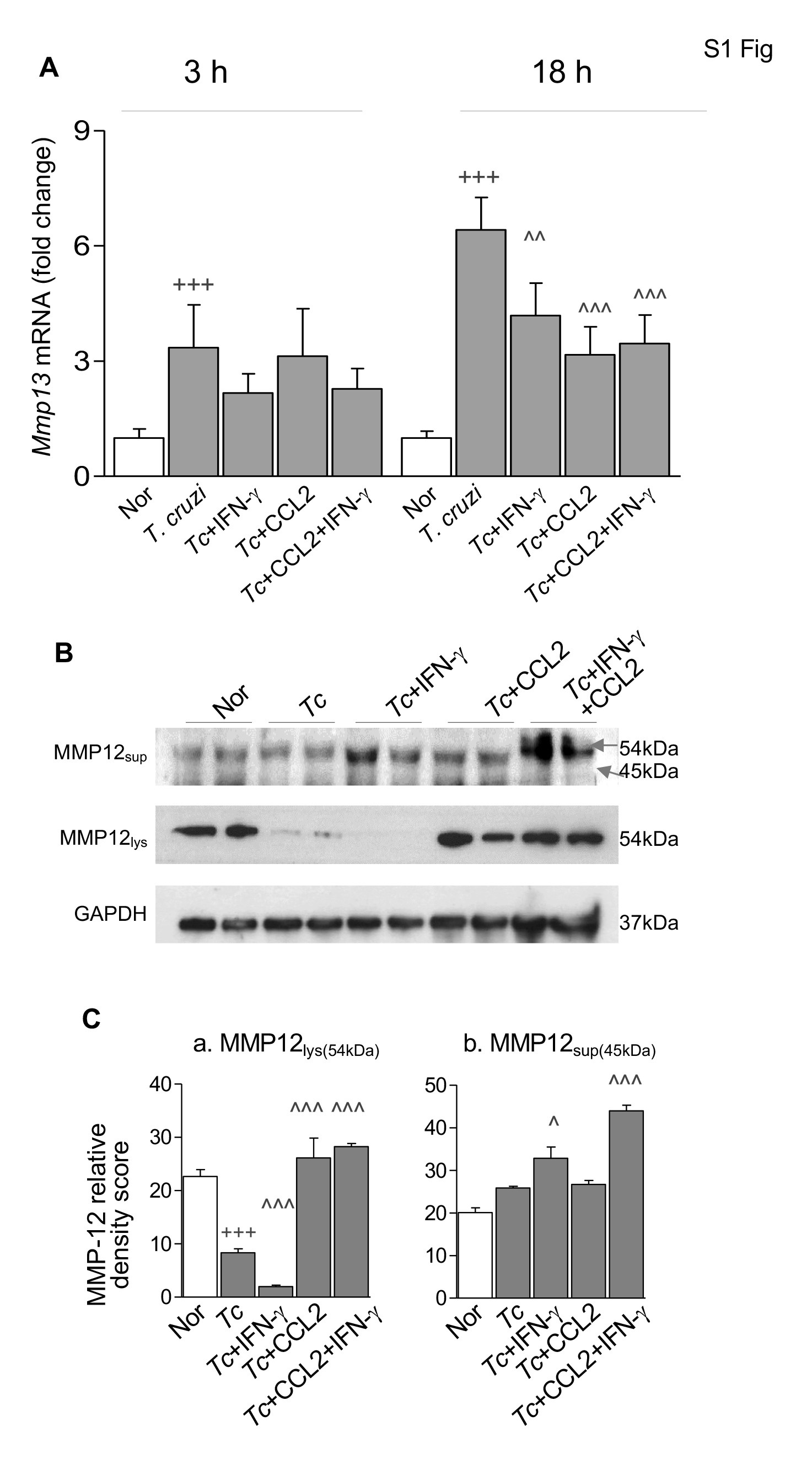

Supplement: FIG S1 [file mBio.01853-20-sf001.tif]

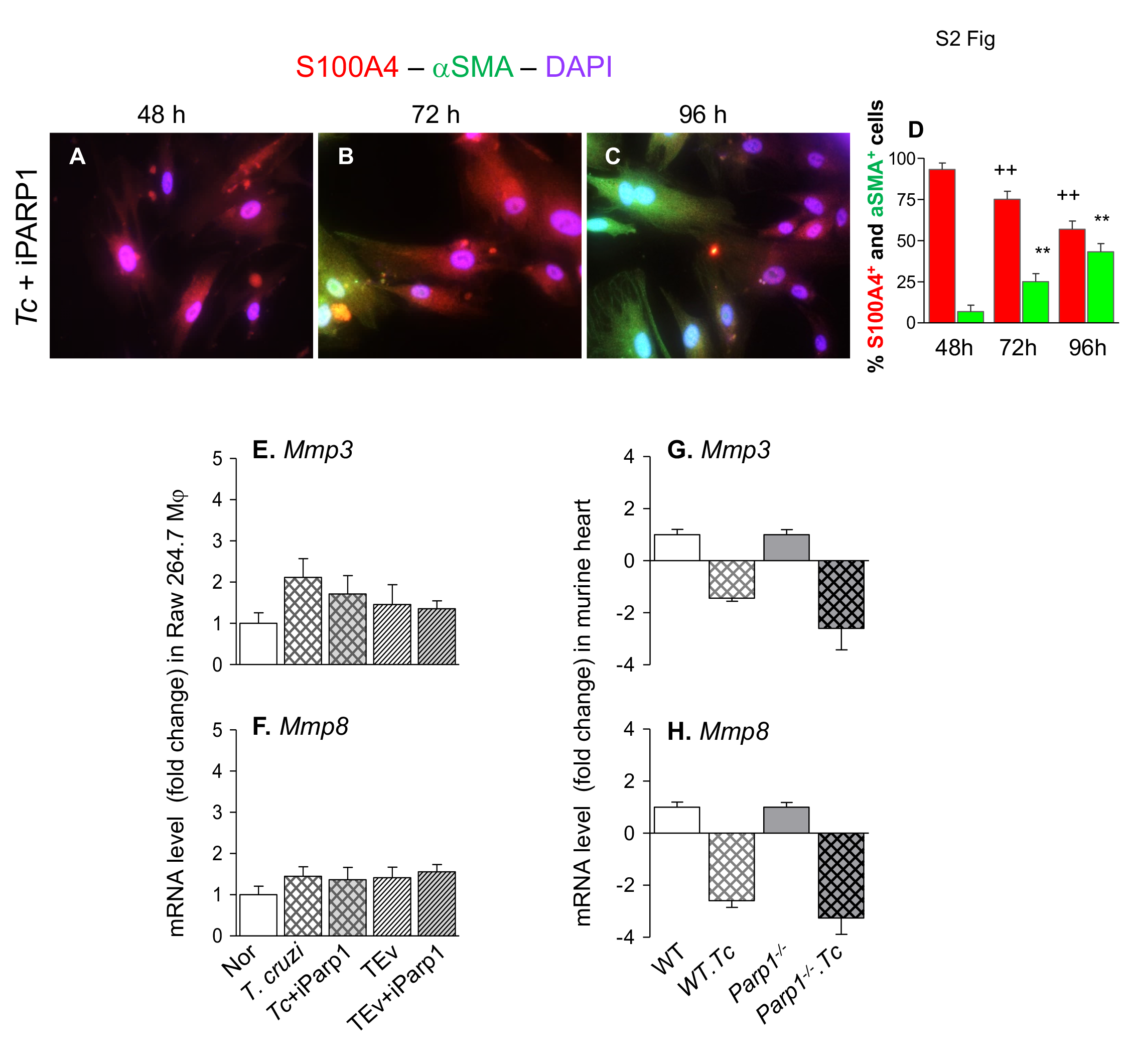

Supplement: FIG S2 [file mBio.01853-20-sf002.tif]

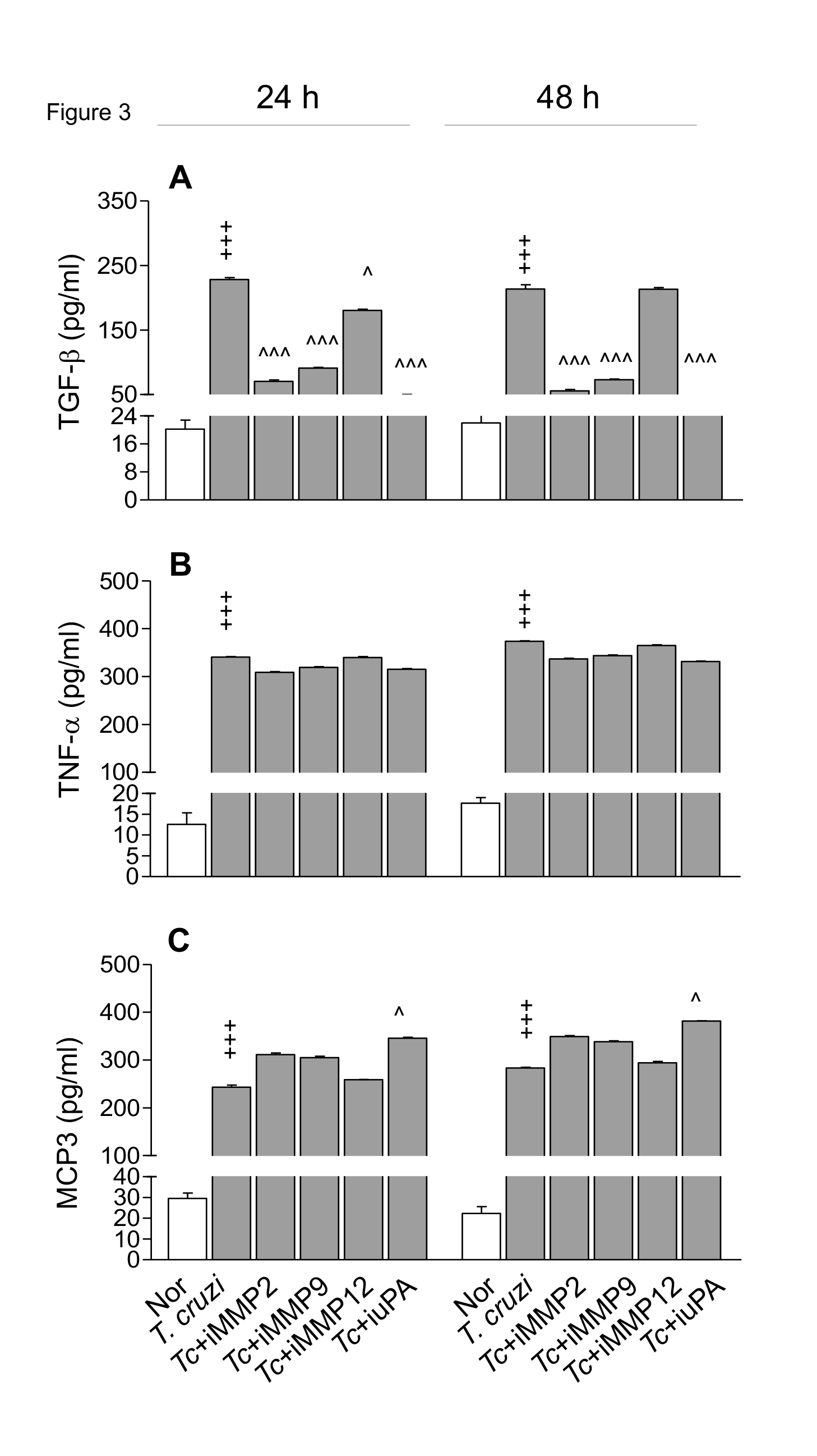

Supplement: FIG S3 [file mBio.01853-20-sf003.tif]
