# Supplementary material for: Trypanosoma cruzi Induces the PARP1/AP-1 Pathway for Upregulation of Metalloproteinases and Transforming Growth Factor β in Macrophages: Role in Cardiac Fibroblast Differentiation and Fibrosis in Chagas Disease
Source: mBio. 2020 Nov 10;11(6):e01853-20. doi: 10.1128/mBio.01853-20 (PMC7667027; doi:10.1128/mBio.01853-20)
Supplement: TEXT S1 [file mBio.01853-20-s0001.docx]

**Supplementary File**

**Detailed Materials and Methods**

**Ethics statement**

All animal experiments were performed by following the NIH guidelines for Care and Use of Experimental Animals, and in accordance with protocols approved by the Institutional Animal Care and Use Committee at the University of Texas Medical Branch, Galveston (protocol number: 0805029).

## Mice, parasites, and cell culture

## The C2C12 immortalized mouse myoblast cells (ATCC CRL-1772) were cultured in complete RPMI 1640 medium containing 5% or 10% FBS. *T*. *cruzi* (SylvioX10/4, ATCC 50823) trypomastigotes were propagated by *in vitro* passage in C2C12 cells.

B6129S/J (Wild type [WT]) and 129S-*Parp1^tm1Zqw^*/J (*Parp1^-/-^*) mice were purchased from Jackson Laboratory (Bar Harbor, ME). The *Parp1* gene deletion in *Parp1^-/-^* mice was confirmed by genotyping for *Parp1* by a standard PCR and by examining *Parp1* mRNA expression by RT-qPCR (1). Six weeks old mice were infected with *T. cruzi* (SylvioX10 strain, 10,000 trypomastigotes per mouse, intraperitoneal) and euthanized at ~150 days post-infection corresponding to chronic disease phase (2).

Raw 264.7 murine Mφ (ATCC TIB-71) were cultured in complete high glucose Dulbecco’s modified Eagle’s medium (DMEM) containing 10% FBS. THP-1 human monocytes (ATCC TIB-202) were incubated at 37^o^C/5% CO_2_ for 24 h in complete RPMI medium containing 10% FBS, 5 mM pyruvate, and 50 ng/mL phorbol 12-myristate 13-acetate (PMA, Sigma-Aldrich, St Louis, MO) to differentiate to mature Mφ, and then incubated for 48 h in complete RPMI media without any stimulus to generate the resting Mφ (3). Human cardiac fibroblasts (HCF, 306-05, Millipore Sigma) were cultured in cardiac fibroblast growth medium (316-500, Millipore Sigma).

## All chemicals used in the study were of molecular grade and purchased from Sigma-Aldrich unless otherwise specified. Fetal bovine serum was heat inactivated at 56^o^C for 30 minutes before use in culture medium.

## Primary macrophages

## Murine bone marrow (BM) cells were isolated from the femurs of WT and *Parp1*^-/-^ mice by following a standard protocol, and either used immediately or stored at -80^o^C in 80% heat-inactivated fetal bovine serum (ha-FBS, Invitrogen, Carlsbad, CA) with 20% DMSO. The BM cells were suspended in RPMI medium containing 10% FBS, 2-mmol/L glutamine, 100 IU/mL penicillin, 100-µg/mL streptomycin (Corning, Corning, NY), added to 6-well plates (5x10^6^ cells/mL/well), and incubated at 37˚C in 5% CO_2_ in presence of 20 ng/mL of macrophage colony stimulating factor (M-CSF; Millipore, Burlington MA). The culture medium and M-CSF were replenished every two days, and cells were incubated for nine days allowing the monocyte progenitor cells to mature as Mφ (4).

Heart tissues obtained from non-infected and chronically-infected mice were subjected to digestion with 40U/mL collagen type II (46H8900, Worthington, NJ, USA) for 1 h. Single cell suspensions of collagen-treated heart tissue and mashed spleen tissue were passed through 70 µM cell strainer, and subjected to Histopaque (10771-100, Sigma-Aldrich) density gradient centrifugation. The mononuclear cells were collected from the opaque interface and CD11b^+^ Mφ were purified by using the MagniSort Mouse CD11b Positive Selection Kit (8802-6860-74, Thermo Fisher Scientific, CA). Purified CD11b^+^ primary Mφ were seeded in 6-well plates (1x10^5^ cells per well) and incubated in complete DMEM/10% FBS for 24 h. Cells and culture supernatants were analyzed for gene expression and cytokines release, respectively.

**Treatment of macrophages**

Raw 264.7, THP-1, or primary Mφ were distributed in 6 or 24-well plates (1-5 X 10^5^ cells/ 2 mL/ well or 1 X 10^4^ cells / 300 µL/well) and incubated for indicated time-points in the presence and absence of *T. cruzi* trypomastigotes (cell-to-parasite ratio, 1: 3), 10 ng/mL JE/MCP-1 (mouse CCL-2, SRP 3215, Millipore Sigma, Burlington, MA), 20 ng/mL IFN-γ (11276905001, Sigma-Aldrich), 20 µM CAS1049741-03-8 (TNF-α inhibitor, 654256-5M, Millipore Sigma), 10 µM iniparib (PARP1 inhibitor, S1087, Selleck Chemicals, Houston, TX), 10 µM ARP-100 (MMP2 inhibitor, sc-203522, Santa Cruz Biotechnology (SCBT), Dallas, TX), 10 µM MMP-9 inhibitor-1 (sc-311437, SCBT), 10 µM MMP-408 (MMP12 inhibitor, 444291-5M, Millipore Sigma), 10 µM uk122 inhibitor of urokinase type plasminogen activator (uPA, sc356185, SCBT), 10 µM SR11302 (AP-1 inhibitor, 2476-10, R&D Systems, Minneapolis, MN), 10 µM JSH-23 (NFκB inhibitor, 481408-M, Millipore Sigma), 10 µM NSC74859 (STAT3 inhibitor, 4655-10, R&D Systems) or 10 µM CADD-522 (RUNX2 inhibitor, HY-107999, MCE, NJ). Cell lysates and supernatants were stored at -20^o^C or at -80^o^C. In some experiments, Mφ were treated with extracellular vesicles (Ev) released by infected cells. For this, Raw 264.7 Mφ were infected with *T. cruzi* (cell: parasite ratio, 1:3) and incubated in serum free RPMI medium at 37^o^C / 5% CO_2_ for 72 h, as described previously ^12^. The culture supernatants were centrifuged at 4000 g for 10 minutes to pellet the cell debris and parasites. Then culture supernatants were subjected to three series of centrifugation at 4^o^C for 30 min each at 20,000 g. The pelleted *T. cruzi*-induced Ev (TEv) from each centrifugation were washed, resuspended at 10-fold concentration in serum free RPMI medium, and stored at -80^o^C. Normal Ev (NEv) harvested from culture medium of uninfected cells were used as controls. Next, a new batch of Raw 264.7 Mφ was seeded in 6-well plates (5x10^6^ cells/well/mL serum free medium). Ev isolated from culture supernatants of non-infected and *T. cruzi*-infected cells were added at 10% media equivalent level, and Mφ were incubated for 3 h and 18 h in the presence and absence of 10 µM iniparib.

## Real time RT-qPCR

Primary or cultured Mφ (± *Tc* infection and/or treatment), CD11b^+^ Mφ isolated from heart and splenic tissues of normal and infected (WT and *Parp1^-/-^*) mice and incubated in duplicate for 24 h (n=3 mice per group per experiment)*,* and heart tissues of normal and infected mice (WT and *Parp1^-/-^*, n=3 mice per group per experiment) were homogenized in Trizol reagent (1-5 x 10^5^ cells, v/v ratio, 1:10). Total RNA was extracted and precipitated by chloroform/isopropanol/ethanol method. Total RNA was treated with RNase-free DNase I (AM2222, Ambion, Austin, TX), and analyzed for quality (OD_260_/OD_280_ ratio > 1.8) and quantity (OD_260_ of 1 = 40 μg/mL) by using a Nano Drop ND-1000 spectrophotometer [[44](#_bookmark39)]. Purified RNA (1 μg) was reverse transcribed using the iScript™ cDNA synthesis kit (1708841, Bio-Rad) and cDNA was diluted 5-fold with nuclease free ddH_2_O. Real time qPCR was performed on an iCycler thermal cycler in a 20-μL reaction containing 2 μl cDNA, 10 μL SYBR green supermix (Bio-Rad), and 20 µM of the gene-specific oligonucleotides. The thermal cycling conditions were 95°C for 3 min and 40 cycles of 95^o^C for 15 sec and 60°C for 30 sec. Specific product amplification was confirmed in the melt curve analysis from 63^°^C to 95^°^C. The PCR base line subtracted curve fit mode was applied for determining the threshold cycle (*C*t), and *C*t values of target mRNAs were normalized to the mean Ct value of *Gapdh* reference cDNA. The relative change in mRNA level of each target gene was calculated by 2^-ΔCt^ [2 ^(-ΔCt sample)^ / 2 ^(– ΔCt of control)^] method ^22^. All oligonucleotides are listed in below.

| **Oligonucleotides used in this study** | | | | | | |
| --- | --- | --- | --- | --- | --- | --- |
| **Gene** | **Protein** | **Accession #** | **Primers** | **Sequence 5'-3'** | **Size (bp)** |  |
| *Gapdh* | GAPDH | NM_001289726.1 | F | AACTTTGGCATTGTGGAAGG | 223 |  |
|  |  |  | R | ACACATTGGGGGTAGGAACA |  |  |
| *Mmp2* | MMP2 | NM_008610 | F | GCGACCACAACCAACTACGA | 99 |  |
|  |  |  | R | TGGCATGGCCGAACTCAT |  |  |
| *Mmp3* | MMP3 | [NM_010809.2](https://www.ncbi.nlm.nih.gov/nucleotide/NM_010809.2?report=genbank&log$=nucltop&blast_rank=1&RID=SA5DZA9B01R) | F | CCCCTGATGTCCTCGTGGTA | 99 |  |
|  |  |  | R | AGGGTGCTGACTGCATCAAAG |  |  |
| *Mmp8* | MMP8 | [NM_008611.4](https://www.ncbi.nlm.nih.gov/nucleotide/NM_008611.4?report=genbank&log$=nucltop&blast_rank=1&RID=SA5PXBG3014) | F | CTCGTGGCTGCTCATGAATTT | 70 |  |
|  |  |  | R | ACATCAAGGCACCAGGATCAG |  |  |
| *Mmp9* | MMP9 | NM_013599 | F | CCTACTGCGGGCTCTTCTGA | 70 |  |
|  |  |  | R | CATCCACATTGCAAGGATTGTC |  |  |
| *Mmp12* | MMP12 | NM_008605 | F | GGGCTGCAGCATTCCAATAA | 90 |  |
|  |  |  | R | GTCATCAGCAGAGAGGCGAAA |  |  |
| *Mmp13* | MMP13 | NM_008607 | F | GAAGACCCCAACCCTAAGCAT | 80 |  |
|  |  |  | R | CGGAGACTGGTAATGGCATCA |  |  |
| *Tgf-β* | **TGF-β** | NM_011577.2 | F | AGGGCTACCATGCCAACTTC | 168 |  |
|  |  |  | R | CCACGTAGTAGACGATGGGC |  |  |

**Gelatin zymography**

Cell culture supernatants obtained from cultured or primary Mφ (± *T. cruzi* and/or treatment) were subjected to zymography to examine the release of MMP2 and MMP9. Briefly, conditioned medium samples (1 mL) were centrifuged at 4000 rpm for 5 minutes to remove the cell debris, incubated with 30 µL gelatin agarose beads at 4^o^C for 1 h, and agarose-bound gelatinases were eluted in 30 µL of 1X sample buffer (5mM Cacl_2_ and 0.02% Brij-35 in 1X Tris buffered saline, pH 7.5 ). Samples (20 µL) were electrophoresed on Novex 10% Zymogram Plus gels (ZY00100BOX, Thermo Fisher Scientific) at 125 V by using an X Cell surelock mini-cell electrophoresis system (Thermo Fisher). Gelatin gels were sequentially incubated in 1X renaturation buffer (LC 2670, Thermo Fisher Scientific) for 1 h and in zymogram developing buffer (LC2671, Thermo Fisher Scientific) for overnight at 37^o^C. Gels were stained with colloidal blue stain containing Coomassie G-250, ammonium sulfate and phosphoric acid ((LC6025, Thermo Fisher Scientific) with 20% methanol (v/v) and 55% deionized water (v/v) for 7-10 h at room temperature with intermittent shaking. After washing with deionized water for 3 h with 2 changes of water, gels were imaged using a Gel Doc EZ System (1708272, Bio-Rad). The areas of destained bands (represent metalloproteinase activity) were measured using Image J software (NIH, Bethesda, MD).

**Western blotting**

## RAW 264.7 Mφ were incubated in presence or absence of *T. cruzi*, inhibitors, or recombinant cytokines as described above and culture supernatants and cells were collected. Cells were lysed in RIPA buffer, centrifuged at 10,000 g and resultant homogenates were used as protein lysates. Protein lysates (20 μg) and culture supernatants were electrophoresed on a 10% polyacrylamide gel by using a Mini-PROTEAN electrophoresis chamber (Bio-Rad), and proteins were transferred to PVDF membrane by using a Criterion Trans-blot System (Bio-Rad). Membranes were blocked with 0.5% BSA in 20 mM Tris-buffered saline (TBS, Tris-HCl pH 7.4 / 136 mM NaCl) containing 0.1% tween 20 (TBST) for 2 h and incubated overnight at 4°C with MMP12 primary antibody (1: 1000 dilution) listed in the table below. Membranes were washed with TBST and TBS, incubated for 1 h at room temperature with HRP-conjugated secondary antibody (1: 5000 dilution, Southern Biotech, Birmingham AL), and color was developed using Pierce ECL Western Blotting Substrate (32106, Thermo Fisher). Images were acquired using an Image Quant LAS4000 system (GE Healthcare, Pittsburgh, MA), protein bands of interest were analyzed by using Image J software, and protein densitometry values were normalized to GAPDH levels.

**Antibodies used in this study**

| **Antigen** | **Cat# (Clone#)** | **Source** | **Host species** |
| --- | --- | --- | --- |
| Matrix metalloproteinase 9 (MMP-9) | Ab38898 | Abcam | Mouse |
| Matrix metalloproteinase 12 (MMP-12) | sc-390863 | Santacruz | Mouse |
| CD68 | ab31630 | Abcam | Mouse |
| S100-A4 | ab93283 | Abcam | Mouse |
| Alpha smooth muscle actin (α – SMA) | ab5694 | Abcam | Rabbit |
| Transforming growth factor beta (TGF-β) | ab92486 | Abcam | Rabbit |
| Galectin 3 | ab53082 | Abcam | Rabbit |
| Vimentin | ab8978 | Abcam | Rabbit |
| Glyceraldehyde 3 P dehydrogenase (GAPDH) | ab9485 | Abcam | Rabbit |

## Enzyme-linked immunosorbent assay (ELISA)

Quantitative ELISA kits (eBiosciences, San Diego, CA) were employed to monitor the release of active TGF-β1(catalogue 88-8350) and TNF-α (catalogue 88-7324) in culture supernatants. MCP3 (CCL7) quantitative ELISA Kit (ab205571, Abcam, MA, USA) was used to monitor MCP3/CCL7 release in culture supernatants. Standard curves were prepared by using recombinant cytokines (4 pg/mL – 10 ng/mL).

**Immunofluorescence**

Human cardio fibroblasts cells were seeded in LabTek II 4 chambers slide (154526, Nunc, Rochester, NY). At 70% confluency, HCF cells were incubated for indicated time points in presence and absence of 20 ng/mL TGF-β1 (ab50036, Abcam, Cambridge, UK) or cell culture supernatants previously obtained from THP-1 Mφ incubated with *T. cruzi* trypomastigotes and various inhibitors, as described above. The culture supernatant was replenished at every 48 h.

After incubation, HCF cells were fixed for 30 min in 4% paraformaldehyde, permeabilized and blocked for 2 h in 1XPBS containing 0.1% Triton X100 / 10% goat serum. Cells were sequentially incubated at 4^o^C for 20 h with anti-mouse S100A4 antibody (1: 50 dilution in 1XPBS / 0.1% Triton X100 / 1% BSA), washed with 1X PBS (5 min each, three times), and labeled for 2 h with Alexa Flour 594-conjugated goat anti-mouse secondary antibody (ab150116, Abcam). Cells were washed again and subjected to staining with rabbit anti-mouse α smooth muscle actin (αSMA) primary antibody (1: 80 dilution in 1XPBS / 0.1% Triton X100 / 1% BSA) and Alexa Flour 488-conjugated goat anti rabbit secondary antibody (ab150077, Abcam) as above. Cells were washed, coverslip-mounted using mounting medium containing the fluorescent nuclear stain 4’,6-diamidino-2-phenylindole (DAPI, Vector Laboratories) and signals were analyzed on an Olympus BX-15 fluorescence microscope (Center Valley, PA) equipped with digital camera and Simple PCI software (v.6.0, Compix, Sewickley, PA) (5).

**Immunohistochemistry**

Slides with paraffin-embedded 5-µm heart tissue sections were deparaffinized, suspended in 0.01 M sodium citrate buffer (pH 6.0) and incubated for 10 min in a boiling water bath to unmask the antigens, and incubated for 10-20 min each with Bloxall blocking solution (Vector Laboratories) and 2.5% normal horse serum to block endogenous peroxidase activity and non-specific antibody binding, respectively. Tissue sections were sequentially incubated for 6-18 h at 4^o^C with primary antibodies against CD68, TGF-β, MMP9, galectin-3 and vimentin (1:50 dilution in PBS containing 1% BSA / 0.1% Triton X100) and for 30 min at room temperature with ImmPRESS Duet Double Detection Reagent (MP-7714, Vector laboratories) containing HRP-conjugated horse anti-rabbit IgG and alkaline phosphatase AP-conjugated horse anti-mouse IgG antibodies . Subsequently, tissue sections were stained with ImmPACT DAB EqV HRP (brown color) and Vector Red AP (magenta color) substrates, fixed in Vecta Mount AQ Aqueous Mounting Medium (H-5501, Vector Laboratories), and imaged at 20X and 60X magnification by using an Olympus BX-15 microscope (Center Valley, PA) equipped with digital camera and Simple PCI software (v.6.0, Compix, Sewickley, PA). Tissue sections (n=3 per group, at least two slides per tissue) were analyzed in at least nine microscopic fields and scored as described previously ^33^. Briefly, distribution of individual antigen was scored as (0) = < 10%, (1^+^) = 10-25%, (2^+^) = 25-50%, (3^+^) = 50-75%, and (4^+^) = > 75% of scanned area. The intensity of staining was scored as (1) = weak, (2^+^) = moderate, and (3^+^) = strong. Tissue dual antigen expression and colocalization analysis was done by semi-quantitative combined scoring system. Tissue slides were scored to capture the percentages of scanned area that were positive for antigen expression (score range 0–4) and exhibited low-to-high intensity of antigen expression (score range: 1–3), and combinative multiplicative score calculated. Finally, an average score was calculated from each combinative score.

**AP-1 transcriptional activity**

BM-derived primary Mφ (WT and *Parp1^-/-^*) were incubated for 3 h and 18 h in the presence or absence of *T. cruzi* and specific inhibitors as described above. Mφ (5 x 10^6^/mL) were lysed on ice for 30 min in buffer A (10 mM HEPES, pH 7.9, 10 mM NaCl, 0.1 mM EDTA, 0.1 mM EGTA, 1 mM DTT, 1 mM PMSF) containing 0.625% NP-40 and 1% protease inhibitor cocktail. Cell lysates were centrifuged at 4^o^C at 10,000 g for 1 min and supernatants stored as a cytosolic fraction at -80^o^C. Pellets were washed with 1 mL buffer A containing 1.7 M sucrose, re-suspended in buffer B (20 mM HEPES pH 7.9, 0.4 M NaCl, 1 mM EDTA, 1 mM EGTA, 1 mM DTT, and 1 mM PMSF), and centrifuged at 4^o^C at 13,000 g for 5 min. The resultant supernatants were stored at −80^o^C as nuclear extracts.

AP-1 transcriptional activity was monitored using the 96-well TransAM AP-1 Family Transcription Factor Assay Kit (44296, Active Motif, CA). Briefly, 5 µg nuclear extracts were diluted in 20 µL lysis buffer, added in duplicate to the 96-well plate coated with oligonucleotide containing TPA-response element (TRE), and incubated for 1 h. The binding of AP-1 dimers contained in nuclear extracts to the immobilized oligonucleotides was detected through incubation with primary antibodies directed against specific components of AP-1 dimers including c-Fos, Fos-B, Fra-1, c-Jun, Jun D and Jun B followed by incubation with HRP-conjugated secondary antibody and TMB substrate, and change in absorbance recorded at 450 nm. The k-562 (TPA) nuclear extract and the mutated oligonucleotide provided in the assay kit were used as positive and negative controls, respectively.

**Statistical analysis**

All experiments were repeated at least twice (2-3 biological replicates analyzed in duplicate or triplicate per experiment). Murine samples (n=3 per group) for histology studies were analyzed in duplicate (9 microscopic fields per tissue section). All data were analyzed by using an InStat version 5 (GraphPad, La Jolla, CA) software and expressed as mean ± standard deviation (SD). To calculate statistical significance, data were analyzed by unpaired Student’s two tailed t-test or Mann Whitney t-test (for comparison of two groups) or one-way analysis of variance (ANOVA) and repeated measure ANOVA with *post hoc* correction test (for comparison of multiple groups). Data are presented as mean ± standard deviation (SD). A p value of < 0.05 was considered as minimum level of significance for the comparison of minimum two variables.

**References**

1. Wen JJ, Yin YW, Garg NJ. 2018. PARP1 depletion improves mitochondrial and heart function in Chagas disease: Effects on POLG dependent mtDNA maintenance. PLoS Pathog 14: e1007065.

2. Wen JJ, Porter C, Garg NJ. 2017. Inhibition of NFE2L2-antioxidant response element pathway by mitochondrial reactive oxygen species contributes to development of cardiomyopathy and left ventricular dysfunction in Chagas disease. Antioxid Redox Signal 27: 550-66.

3. Dey N, Sinha M, Gupta S, Gonzalez MN, Fang R, et al. 2014. Caspase-1/ASC inflammasome-mediated activation of IL-1beta-ROS-NF-kappaB pathway for control of *Trypanosoma cruzi* replication and survival is dispensable in NLRP3^-/-^ macrophages. PLoS One 9: e111539.

4. Koo SJ, Szczesny B, Wan X, Putluri N, Garg NJ. 2018. Pentose phosphate shunt modulates reactive oxygen species and nitric oxide production controlling *Trypanosoma cruzi* in macrophages. Front Immunol 9: 202.

5. Choudhuri S, Garg NJ. 2020. PARP1-cGAS-NFkB pathway of proinflammatory macrophage activation by extracellular vesicles released during *Trypanosoma cruzi* infection and Chagas disease. Plos Pathog 16(4): e1008474.
